# CD11c^+^ B Cells Participate in the Pathogenesis of Graves’ Disease by Secreting Thyroid Autoantibodies and Cytokines

**DOI:** 10.3389/fimmu.2022.836347

**Published:** 2022-03-21

**Authors:** Yedi Cao, Xue Zhao, Ran You, Yang Zhang, Chenxue Qu, Youyuan Huang, Yang Yu, Yan Gong, Tiechuan Cong, Enmin Zhao, Lanbo Zhang, Ying Gao, Junqing Zhang

**Affiliations:** ^1^ Department of Endocrinology, Peking University First Hospital, Beijing, China; ^2^ Department of Clinical Laboratory, Peking University First Hospital, Beijing, China; ^3^ Department of Otolaryngology-Head and Neck Surgery, Beijing, China; ^4^ Breast Disease Center, Peking University First Hospital, Beijing, China

**Keywords:** CD11c^+^ B cells, TRAb, Graves’ disease, cytokines, CXCR3-CXCL10

## Abstract

Graves’ disease (GD) is a common autoimmune disorder with an elevation in pathogenic autoantibodies, specifically anti-thyrotropin receptor antibodies (TRAbs), which are secreted by autoreactive B cells. To date, there has been little research on self-reactive B cells in GD. In the current study, we reported that a unique B-cell subset, CD11c^+^ B cells, was expanded in the peripheral blood (PB) of GD patients, as detected by flow cytometry. The frequency of CD11c^+^ B cells was positively correlated with serum TRAb levels. The flow cytometry data showed that CD11c expression was higher in a variety of B-cell subsets and that CD11c^+^ B cells presented a distinct immunophenotype compared to paired CD11c^-^ B cells. Immunohistochemical and immunofluorescence staining indicated the presence of CD11c^+^CD19^+^ B cells in lymphocyte infiltration areas of the GD thyroid. Flow cytometric analysis of PB and fine-needle aspiration (FNA) samples showed that compared to PB CD11c^+^ B cells, CD11c^+^ B cells in the thyroid accumulated and further differentiated. We found that CD11c^+^ B cells from the PB of GD patients were induced to differentiate into autoreactive antibody-secreting cells (ASCs) capable of secreting TRAbs *in vitro*. Luminex liquid suspension chip detection data showed that CD11c^+^ B cells also secreted a variety of cytokines, including proinflammatory cytokines, anti-inflammatory cytokines, and chemokines, which might play roles in regulating the local inflammatory response and infiltration of lymphocytes in the thyroid. In addition, we performed a chemotaxis assay in a Transwell chamber to verify that CD11c^+^ B cells were recruited by thyroid follicular cells (TFCs) *via* the CXCR3-CXCL10 axis. In conclusion, our study determined that CD11c^+^ B cells were involved in the pathogenesis of GD in multiple ways and might represent a promising immunotherapeutic target in the future.

## Introduction

Graves’ disease (GD), as the most common cause of persistent hyperthyroidism in adults (1), is an organ-specific autoimmune disease characterized by diffuse goiter and an elevation in anti-thyrotropin receptor antibodies (TRAbs). Some patients also present with extrathyroidal complications, such as Graves’ orbitopathy (GO), and untreated hyperthyroidism is related to increased risks of osteoporosis (2), fracture (3), stroke (4), and cardiovascular events (5, 6). For the past 70 years, conventional treatments for GD, including pharmacotherapy with antithyroid drugs (ATDs), radioiodine (RAI) therapy, and surgery, have remained largely unchanged despite many patients exhibiting a substantial unmet clinical need (1, 7–10). New therapeutic options need to be combined with greater insight into the autoimmune pathogenesis of GD.

GD is commonly considered an archetypal B-cell-mediated autoimmune disorder, occurring *via* a breach in tolerance that allows autoreactive B cells to be activated and expand at disease onset (11). Circulating TRAbs, as pathogenic autoantibodies, are secreted by self-reactive B cells and behave most often as thyroid-stimulating antibodies to activate the thyrotropin receptor in thyroid follicular cells (TFCs), leading to hyperthyroidism (12, 13). Thus, the elimination of autoreactive B cells may be an ideal therapy to inhibit the production of TRAbs in GD patients. To date, there is a paucity of research focusing specifically on autoreactive B cells in GD.

A growing body of evidence shows that CD11c^+^ B cells, as autoreactive B cells, have been observed to expand in various autoimmune diseases (14–17). Transcriptome and IgH repertoire analyses have identified CD11c^+^ B cells as a unique B-cell subset distinct from naïve, memory, and plasma cells (18). CD11c^hi^ B cells isolated from the peripheral blood (PB) of systemic lupus erythematosus (SLE) patients were found to be poised to differentiate into plasma cells and produce the majority of autoantibodies. The expansion of CD11c^hi^ B cells in SLE was associated with submanifestations and disease activity scores (19). Previous studies have shown that the phenotype and frequency of CD11c^+^ B cells are heterogeneous among different individuals and diseases (20). Therefore, the pathogenicity of CD11c^+^ B cells in GD patients requires further investigation, as these cells may play critical roles in the onset and development of GD.

In the current study, we first confirmed the expansion of CD11c^+^ B cells in GD patients and characterized the phenotype of CD11c^+^ B cells in both the PB and thyroid of GD patients. Furthermore, we evaluated the TRAb production and cytokine profiles of PB CD11c^+^ B cells from GD patients. We found that CD11c^+^ B cells were recruited by TFCs *via* the CXCR3-CXCL10 axis. All the results indicated that autoreactive CD11c^+^ B cells were involved in the development of GD, which might represent a novel immunotherapeutic target in GD.

## Materials and Methods

### Subjects and Sample Collection

All participants were enrolled in this study after informed consent was obtained; this study was conducted from May 2019 to October 2021 at Peking University First Hospital. Seventy-one GD patients (including 23 GO patients) diagnosed according to the 2016 American Thyroid Association (ATA) guidelines (21) and the 2021 European Group on Graves’ Orbitopathy (EUGOGO) clinical practice guidelines (22) were enrolled in the current study. Forty-two healthy donors (HD) were euthyroid and negative for thyroid autoantibodies, including anti-thyroglobulin antibodies (TgAbs), anti-thyroid peroxidase antibodies (TPOAbs) and TRAbs, and they had no family history of thyroid diseases or relevant medical history. Participants were excluded from this study if they had any other autoimmune disorder or coexisting malignancy or were undergoing immunosuppressive drug or steroid treatment.

Human whole-blood samples were collected from all the participants. One hundred microliters of PB was taken for flow cytometry detection, and the remaining blood pellets were immediately used for cell sorting.

Samples collected by fine-needle aspiration (FNA) were obtained from thyrotoxic patients who underwent fine-needle aspiration cytology (FNAC) to determine the etiology of thyrotoxicosis (23–26). A total of 10 patients were diagnosed with GD.

Surgical thyroid tissue samples were obtained from another 4 GD patients undergoing subtotal thyroidectomy at Peking University First Hospital. The tissue samples were washed with phosphate-buffered saline (PBS), immediately fixed with formalin and embedded in paraffin.

The study complied with the Helsinki Declaration; it was approved by the Ethics Committee of Peking University First Hospital and conducted in accordance with approved guidelines. Witten informed consent was obtained from all participants in this study (2021-318).

### Laboratory Testing of Thyroid Function and Thyroid Autoantibodies in GD Patients and HD

The levels of TSH, free triiodothyronine (fT3), free tetraiodothyronine (fT4) [ADVIA Centaur (Siemens Healthcare Diagnostics, USA)], TRAbs, TgAbs and TPOAbs [Cobas e601 analyzer (Roche Diagnostics, Switzerland)] were detected by chemiluminescence immunoassays.

### Flow Cytometric Analysis

PB was collected from GD patients (n=71) and HD (n=42) and washed for single-cell isolation for flow cytometry. For cell surface staining, a total volume of 100 μl of single-cell suspension was stained for surface markers in round-bottom tubes for 30 min at room temperature (RT). If intracellular staining (T-bet staining) was performed, cells were then fixed and permeabilized with the True-Nuclear™ Transcription Factor Buffer Set (BioLegend, USA) according to the manufacturer’s instructions. The fluorochrome-conjugated anti-human antibodies were diluted and added to samples at RT for 20 min. All detailed information on the antibodies is provided in **Supplementary Table S1**. Isotype-matched antibodies were used as negative controls. The above antibodies were obtained from BioLegend, Inc., unless otherwise indicated. After staining, the cell pellets were washed twice and resuspended in 500 μl of PBS for flow cytometric analysis. All flow cytometry experiments were performed on a FACSCanto II flow cytometer and were analyzed with FlowJo software (Version 10, FlowJo, USA).

All FNA samples (n=10) from GD patients were immediately washed with PBS and passed through 40-μm cell strainers to remove potential clumps for flow cytometry assays according to the methods described above.

### Immunohistochemical Staining for Human CD11c, CD19 and CXCL10 in GD Thyroid Tissues

Formalin-fixed paraffin-embedded (FFPE) blocks of GD thyroid tissues (n=4) were cut into 4-μm serial sections for immunohistochemical staining. Immunostaining for human CD11c (at a 1:300 dilution, Abcam, Cambridge, UK), human CD19 (at a 1:400 dilution, Abcam, Cambridge, UK), or CXCL10 (at a 1:200 dilution, Proteintech, China) was performed by 3,3’-diaminobenzidine (DAB) staining (Zhongshan Jinqiao Biotechnology, Beijing, China) according to the manufacturer’s instructions. CD19 antigen repair was conducted using citric acid solution (pH=6.0) for 15 min by microwave heating. CD11c and CXCL10 antigen repair was performed with Tris–EDTA buffer and heated for 15 min in a microwave. Two sections were prepared as negative controls for each immunostaining batch.

### Multiplex Immunofluorescence Staining of GD Thyroid Tissues

FFPE GD thyroid tissues (n=4) were cut into 4-μm sections for immunofluorescence staining. Multiplex immunofluorescence staining was performed by using a PANO 4-plex IHC kit (Panovue, China) according to the manufacturer’s instructions. Briefly, sections were deparaffinized and then subjected to antigen repair. The antigen repair conditions were the same as immunohistochemical staining for human CD11c and CD19. Then, primary antibodies were sequentially applied after blocking, followed by HRP-conjugated antibody incubation. The details and usage of primary antibodies and the fluorescent dye in kit were provided in **Supplementary Table S2**. After each TSA staining round, the slides were heated in a microwave to remove all primary and detection antibodies. Finally, 4’-6’-diamidino-2-phenylindole (DAPI; Sigma–Aldrich, USA) was used to stain cell nuclei.

### Cell Sorting of Total B Cells, CD11c^+^ B Cells, and CD11c^-^ B Cells From PB Samples With Immunomagnetic Beads

To detect the functions of total B cells, CD11c^+^ B cells, and CD11c^-^ B cells from GD patients, remaining whole human PB samples were harvested after density gradient centrifugation with Ficoll-Paque PLUS (GE Life Sciences, USA), and peripheral blood mononuclear cells (PBMCs) were separated for subsequent magnetic bead sorting. B cells were isolated using the MojoSort™ Human B Cell (CD43-) Negative Isolation Kit (BioLegend, USA) according to the manufacturer’s protocol. Purified B cells were further stained with PE-conjugated anti-CD11c at 4°C in the dark for 30 min, and then CD11c^+^ B cells and CD11c^-^ B cells were sorted by using MojoSort™ Human anti-PE Nanobeads (BioLegend, USA). The purity of sorted B cells was detected on a FACSCanto II flow cytometer.

### Differentiation of B Cells Into Antibody-Secreting Cells (ASCs) *In Vitro*

To investigate the IgG secretion of total B cells, CD11c^+^ B cells, and CD11c^-^ B cells from the PB of GD patients, we established a controlled culture system to induce B cells to differentiate into ASCs. B cells, CD11c^+^ B cells, and CD11c^-^ B cells isolated from the PB of GD patients were cultured separately at a density of 1×10^5^ cells per well in a total volume of 200 μl in 96-well round-bottom plates. A TLR7 agonist (1 µg/ml, R848, resiquimod, *In vivo*Gen, USA) and 100 U/ml IL-2 (PeproTech, USA) were added to costimulate the cells to drive the extrafollicular B-cell response for 9-12 days (27). For continuous detection of total IgG in the culture supernatant, the differentiation culture was extended up to 12 days, and the culture supernatant was collected for subsequent detection. The percentage of plasmablasts was detected by flow cytometry according to the procedure described above. The culture medium of the B-cell differentiation culture experiment was RPMI 1640 medium supplemented with 10% FBS, 100 U/ml penicillin, 100 U/ml streptomycin, 2 mM L-glutamine (all from Gibco, USA), and Insulin Transferrin Selenium (ITS; 1.0 mg/ml insulin, 1.0 mg/ml transferrin, 3.4 µM selenium, ScienCell, USA). Experiments were performed at least three times with different donors.

### Measurement of IgG and TRAb Concentrations in Culture Supernatants *In Vitro*

The ELISA double-antibody sandwich method was performed to measure the titers of IgG secreted by B cells, CD11c^+^ B cells, and CD11c^-^ B cells from the PB of GD patients into culture supernatants as previously described (28, 29). Briefly, a 96-well plain plate was precoated with a human IgG capture antibody (1:400 dilution, Abcam, UK). After blocking with 3% bovine serum albumin (BSA, Sigma–Aldrich), the culture supernatant at the optimal dilution was added to the plate. HRP-conjugated anti-human IgG (1:2000, Abcam, UK) was incubated with the sample and reacted with O-phenylenediamine (OPD; Sigma–Aldrich, USA). The absorbance at 492 nm was determined with a microplate reader, and the titers of IgG were calculated based on the absorbance readings of the IgG standard curve.

The titer of TRAbs in culture supernatants was measured with the Human TSHR-Ab (Anti-Thyroid Stimulating Hormone Receptor) ELISA Kit (Elabscience, China) according to the manufacturer’s instructions. In brief, undiluted samples were added to precoated wells and incubated with a capture antibody for 90 min at 37°C. The wells were aspirated and then treated with biotinylated TSHR. Then, streptavidin-HRP was added to the plate, and 3,3’,5,5’-tetramethylbenzidine (TMB), as the substrate, was added to the wells and allowed to react for 20 min. Following the addition of a stop solution, the absorbance at 450 nm was recorded with a microplate reader.

### Luminex Liquid Suspension Chip for Detection of the Cytokine Secretion Profile in Culture Supernatants

To determine the difference in cytokine profiles between CD11c^+^ B cells and CD11c^-^ B cells, five batches of paired CD11c^+^ B cells and CD11c^-^ B cells from 15 GD patients were seeded in a round-bottom 96-well plate at 2×10^5^ cells/well. Then, the sorted B cells were activated with 10 µg/ml *Phaseolus vulgaris agglutinin* (PHA; Sigma–Aldrich, USA) and cultured in a 37°C incubator with 5% CO_2_ for 72 h. Culture supernatant samples for the paired CD11c^+^ B cells and CD11c^-^ B cells were collected and stored at -80°C for Luminex liquid suspension chip detection, which was conducted by Wayen Biotechnologies (Shanghai, China). The Bio-Plex Pro Human Cytokine Grp I Panel 27-plex (Bio–Rad, USA) was used to quantify the concentrations of cytokines secreted by CD11c^+^ B cells and CD11c^-^ B cells from GD patients according to the manufacturer’s instructions. The lower limits of detection (LLOD) of all cytokines in the Luminex liquid suspension chip are shown in **Supplementary Table S3**.

### Transwell Chamber to Evaluate the Chemotactic Ability of CD11c^+^ B Cells

To investigate whether CD11c^+^ B cells from GD patients were recruited by TFCs, the thyroid gland epithelial cell line Nthy-ori3-1 stimulated with IFN-γ was used to mimic the thyroid microenvironment of recent-onset GD (30, 31). Nthy-ori3-1 cells were obtained from the Cell Bank of the Chinese Academy of Sciences (Shanghai, China) and authenticated at Genetic Testing Biotechnology Corporation (Suzhou, China). Nthy-ori3-1 cells were cultured in RPMI 1640 medium supplemented with 10% FBS and 100 U/ml penicillin and streptomycin and seeded in a 12-well plate at a density of 1×10^5^ cells/ml. Then, 1000 U/ml IFN-γ (PeproTech, USA) was added to stimulate the Nthy-ori3-1 cells for 72 h at 37°C in 5% CO_2_. The culture supernatants were centrifuged to remove cells and debris and collected to measure the concentration of CXCL10 by Human IFN gamma Uncoated ELISA (Invitrogen, USA) according to the manufacturer’s instructions. The remaining samples were stored at -80°C.

Twenty-four-well Transwell chambers (Corning, USA) with 8-μm-pore filters were used to analyze the migration of B cells from GD patients. A total of 1×10^5^ B cells, CD11c^+^ B cells or CD11c^-^ B cells in 200 μl of culture medium were added to the upper chamber. Then, 600 μl of supernatant from a Nthy-ori3-1-cell culture stimulated with IFN-γ was added to the bottom chamber to mimic the thyroid microenvironment of GD. After 3 h of migration, the cells in both the upper and lower chambers were collected, counted, and evaluated by flow cytometry according to the protocol described above. Experiments were performed at least three times with different donors.

### Statistical Analysis

All statistical analyses were performed with GraphPad Prism 8.0 (GraphPad Software Inc., USA). Normally distributed data are expressed as the mean ± standard deviation (SD); otherwise, data are expressed as the median and interquartile range (IQR), as appropriate. Count data were analyzed with the chi-square test. Continuous variables with a normal distribution were assessed with a paired or unpaired Student’s t test for comparisons between two groups and with ANOVA for multigroup comparisons. For nonnormally distributed data, nonparametric Mann–Whitney U tests were used for two-group comparisons, and the Kruskal–Wallis test was used for multigroup comparisons. Correlation analyses were performed using the Spearman correlation test for nonparametric distributions. *P <*0.05 was considered statistically significant.

## Results

### Expansion of CD11c^+^ B Cells in GD Patients Compared to HD Patients

We first compared the demographic and clinical characteristics of GD patients and HD. As shown in **Table 1**, the two groups were well matched with regard to age (*P*=0.891) and sex (*P*=0.173) distributions. The levels of thyroid autoantibodies, including TRAbs, TPOAbs, and TgAbs, were significantly higher in GD patients than in HD patients. In regard to thyroid function, significantly higher levels of fT4 and a lower level of TSH were observed in GD patients than in HD patients (*P*<0.001).

**Table 1:** Demographic and clinical characteristics of healthy donors and Graves’ disease patients.

Group	Healthy Donors	Graves’ disease	p value
n	42	71	–
Sex M/F	9/33	16/55	0.891
Age (years)	49.0 (37.5-57.3)	50.11 (35.00-62.00)	0.173
fT4 (pmol/L)	12.42 (10.86-14.03)	15.78 (13.36-20.30)	<0.001
fT3 (pmol/L)	4.87 (4.58-5.19)	5.12 (4.48-6.73)	0.135
TSH (μIU/ml)	2.27 (1.65-3.64)	0.38 (0.01-1.22)	<0.001
TRAb (IU/ml)	0.3 (0.30-0.35)	7.34 (2.59-14.96)	<0.001
TPOAb (IU/ml)	6.15 (5.00-8.50)	108.10 (23.24-278.1)	<0.001
TgAb (IU/ml)	10.00 (10.00-10.55)	107.30 (15.48-483.10)	<0.001

fT4, free tetraiodothyronine; fT3, free triiodothyronine; TSH, thyroid-stimulating hormone; TRAb, anti-thyrotropin receptor antibody; TgAb, anti-thyroglobulin antibody; TPOAb, anti-thyroid peroxidase antibody.

Nonnormally distributed data are expressed as the median and IQR. The counting data were analyzed by a chi-square test. Continuous variables with nonnormal distributions were assessed with Mann–Whitney U tests. P <0.05 was considered statistically significant.

**Figure 1:**
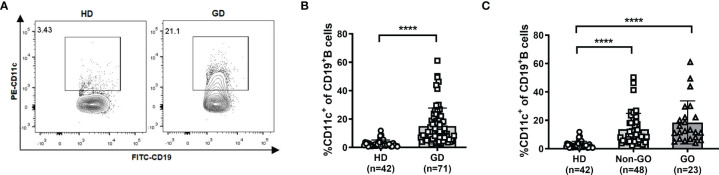
Frequency of CD11c^+^ B cells in healthy donors (HD) and Graves’ disease (GD) patients. **(A)** Representative flow cytometric plots of HD and GD patients. **(B)** Frequency of CD11c^+^ B cells in CD19^+^ B cells of HD and GD patients. **(C)** Frequency of CD11c^+^ B cells in CD19^+^ B cells of HD, non-Graves’ ophthalmopathy (non-GO) patients, and GO patients. Nonnormally distributed data are expressed as the median and IQR, assessed by Mann–Whitney U tests. *P < *0.05 was considered statistically significant. ns, not significant; ****P < 0.0001.

The gating strategy for CD19^+^ B cells and CD11c^+^ B cells is shown in **Supplementary Figure S1**. In the PB, no significant difference in the CD19^+^ B-cell frequency was observed between GD patients and HD (**Supplementary Figure S2**). Strikingly, the frequency of CD11c^+^ B cells in PBMCs was significantly increased in GD patients (*P*<0.001; **Figures 1A, B**), which indicated that the expansion of this abnormal B-cell subset rather than the expansion of total B cells was associated with the occurrence of GD. We further compared the frequency of CD11c^+^ B cells among HD, non-GO patients, and GO patients and found that CD11c^+^ B cells were significantly expanded in both non-GO and GO patients compared to HD (*P*<0.001) (**Figure 1C**). No correlation was found between the frequency of CD11c^+^ B cells and age in GD patients (**Supplementary Figure S3**).

### CD11c^+^ B Cells From the PB of GD Patients Were Correlated With the Levels of Thyroid Autoantibodies

**Figure 2:**
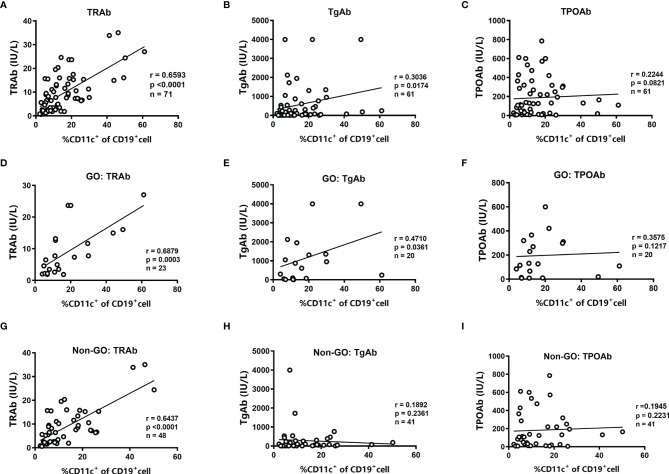
Analysis of the correlations between the frequency of CD11c^+^ B cells and the levels of thyroid autoantibodies. **(A–C)** Linear correlation analysis between the levels of anti-thyrotropin receptor antibodies (TRAbs), anti-thyroglobulin antibodies (TgAbs), or anti-thyroid peroxidase antibodies (TPOAbs) and the frequency of CD11c^+^ B cells in CD19^+^ B cells for all enrolled GD patients. **(D–F)** Linear correlation analysis between the titer of TRAbs, TgAbs, or TPOAbs and the frequency of CD11c^+^ B cells in CD19^+^ B cells for enrolled GO patients. **(G–I)** Linear correlation analysis between the titer of TRAbs, TgAbs, or TPOAbs and the frequency of CD11c^+^ B cells in CD19^+^ B cells for enrolled non-GO patients. The correlation analyses above were performed using the Spearman correlation test. *P <* 0.05 was considered statistically significant.

We further analyzed the relationships between the frequency of CD11c^+^ B cells and the levels of thyroid autoantibodies. In all GD patients, we found that the frequency of CD11c^+^ B cells showed a significantly positive correlation with the titer of TRAbs (r=0.66, P<0.001) and a weak positive correlation with the titer of TgAbs (r=0.30, *P*=0.017). No association was found with the TPOAb titer (**Figures 2A–C**). In patients with GO, we found that the levels of both TRAbs and TgAbs were positively related to the frequency of CD11c^+^ B cells (r=0.69 for TRAbs, *P*<0.001; r=0.47 for TgAbs, *P*=0.036), but no association was identified between the TPOAb level and CD11c^+^ B-cell frequency (**Figures 2D–F**). In the group of non-GO patients, a strong correlation was observed only between the titer of TRAbs and the frequency of CD11c^+^ B cells (r=0.64, *P*<0.0001) (**Figures 2G–I**). In addition, no correlations were found between thyroid function and the frequency of CD11c^+^ B cells (**Supplementary Figure S4**).

### CD11c Was Expressed at Higher Levels in a Variety of PB Memory B-Cell Subsets and ASCs in Both GD and HD Groups

**Figure 3:**
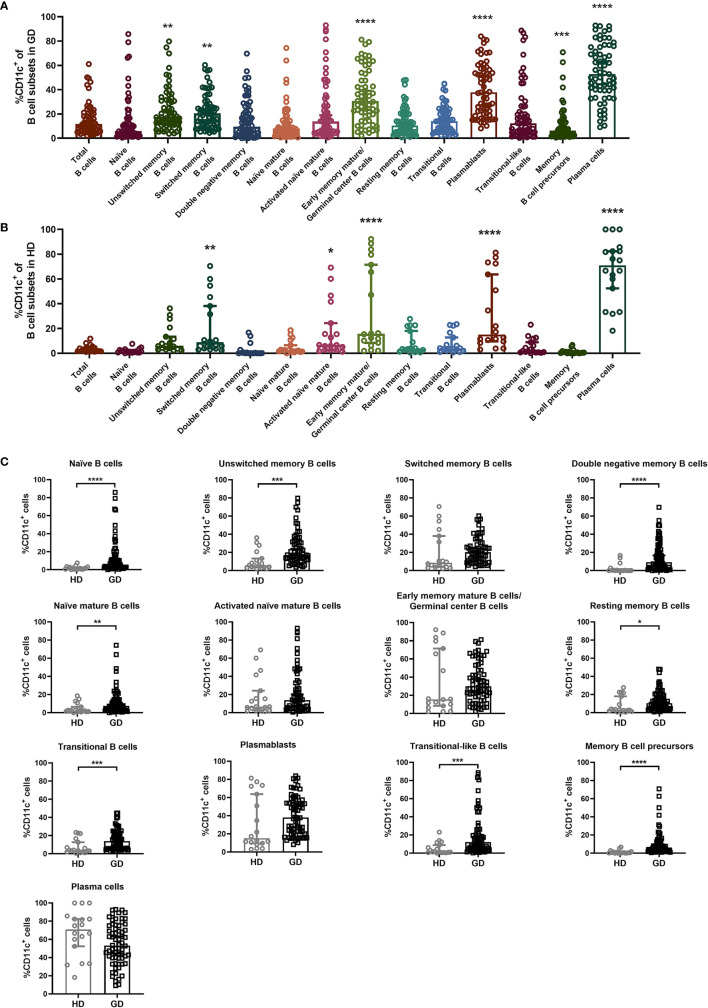
Expression distribution of CD11c in a variety of B-cell subsets. **(A, B)** Total CD19^+^ B cells were divided into 13 B-cell subsets according to the expression of IgD, CD27, CD38, and CD138: naïve B cells (CD27^-^IgD^+^), unswitched memory B cells (CD27^+^IgD^+^), switched memory B cells (CD27^+^IgD^-^), double negative memory B cells (CD27^-^IgD^-^), naïve mature B cells (CD38^-^IgD^+^), activated naïve mature B cells (CD38^+^IgD^+^), early memory mature B cells/germinal center B cells (CD38^+^IgD^-^/CD38^high^IgD^-^), resting memory B cells (CD38^-^IgD^-^), transitional B cells (CD38^-^CD27^+^), plasmablasts (CD38^+^CD27^+^), transitional-like B cells (CD38^+^CD27^-^), memory B-cell precursors (CD38^-^CD27^-^), and plasma cells (CD138^+^). Comparison of the frequency of CD11c^+^ B cells in the above 13 B-cell subsets in 68 GD patients and 18 HD patients. Nonnormally distributed data are expressed as the median and IQR, assessed by the Kruskal–Wallis multiple comparison test. *P <* 0.05 was considered statistically significant. *compared to total B cells, *P <* 0.05; ** compared to total B cells, *P <* 0.01; *** compared to total B cells, *P < *0.001; **** compared to total B cells, *P < *0.0001. **(C)** The comparison of CD11c^+^ B-cellcell frequency in 13 kinds of B-cell subsets between the HD group and GD group. Nonnormally distributed data are expressed as the median and IQR, assessed by Mann–Whitney U tests. *P <* 0.05 was considered statistically significant. *P < 0.05; **P < 0.01; ***P < 0.001; ****P < 0.0001.

To analyze the expression distribution of CD11c in various B-cell subsets in both GD and HD groups, B cells were first gated by CD19, and then the expression of IgD, CD27, CD38 and CD138 allowed the distinction of thirteen B-cell subsets (**Supplementary Figure S5**), including naïve B cells (CD27^-^IgD^+^), unswitched memory B cells (CD27^+^IgD^+^), switched memory B cells (CD27^+^IgD^-^), double negative memory B cells (CD27^-^IgD^-^) (32), naïve mature B cells (CD38^-^IgD^+^), activated naïve mature B cells (CD38^+^IgD^+^), early memory mature B cells/germinal center B cells (CD38^+^IgD^-^/CD38^high^IgD^-^), resting memory B cells (CD38^-^IgD^-^) (33), transitional B cells (CD38^-^CD27^+^), plasmablasts (CD38^+^CD27^+^) (32), transitional-like B cells (CD38^+^CD27^-^) (34), memory B-cell precursors (CD38^-^CD27^-^) (35, 36), and plasma cells (CD138^+^) (37). Compared to total CD19^+^ B cells, CD11c was expressed at higher levels in unswitched memory B cells, switched memory B cells, early memory mature B cells/germinal center B cells, plasmablasts and plasma cells in GD patients (**Figure 3A**). In the HD group, CD11c in the B-cell subset showed a similar expression distribution to that in GD patients (**Figure 3B**). This result indicated that CD11c^+^B cells may share a similar qualitative distribution between GD patients and healthy donors, which is consistent with the results found in SLE and primary Sjögren’s syndrome (pSS) (17). Furthermore, we compared the frequency of CD11c+ cells in various B-cell subsets between the HD and GD groups. Our data showed that a higher frequency of CD11c^+^ B cells was found in some B subpopulations, including naïve B cells, unswitched memory B cells, double-negative memory B cells, naïve mature B cells, resting memory B cells, transitional B cells, transitional-like B cells, and memory B-cell precursors in GD (**Figure 3C**).

### Phenotypes of CD11c^+^ B Cells in the PB of GD Patients

**Figure 4:**
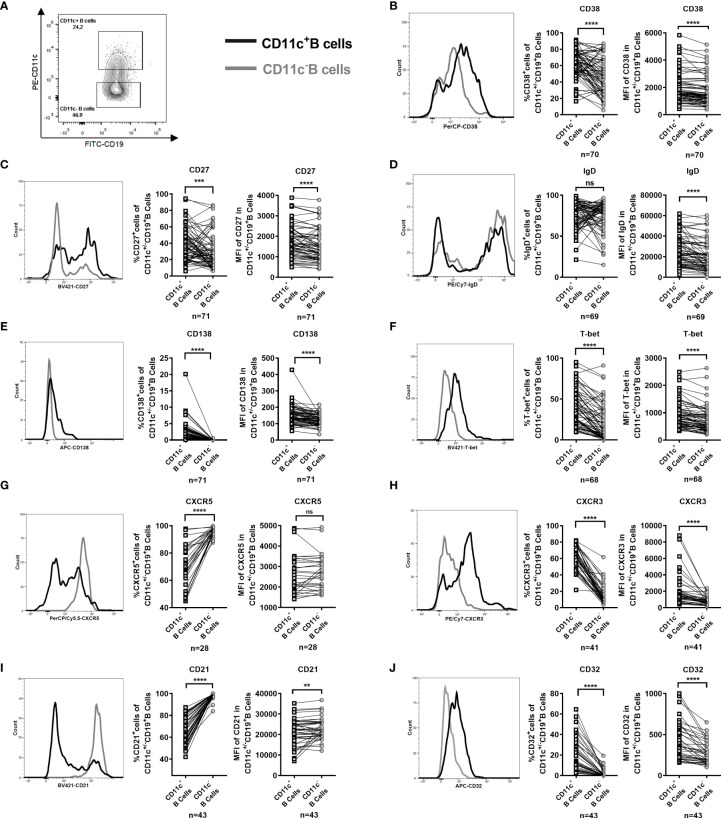
Phenotype of paired CD11c^+^ B cells and CD11c^-^ B cells in the peripheral blood (PB) of GD patients. **(A)** Representative flow cytometric plots of CD11c^+^ B cells and CD11c^-^ B cells in the PB of GD patients. **(B–J)** Comparative overlapped histograms of the PB example in CD11c^+^ and CD11c^-^ B cells for compared markers (left). Comparison between the frequency of subsets of CD11c^+^ B cells and that of paired CD11c^-^ B cells in each PB sample (middle). Comparison between the mean fluorescence intensities (MFIs) of the representative phenotype of CD11c^+^ B cells and that of paired CD11c^-^ B cells in each PB sample (right). The comparison metrics included conventional antigens (CD38, CD27, IgD, and CD138), an unconventional antigen (T-bet), chemokine receptors (CXCR3 and CXCR5), and autoimmune-related antigens (CD32 and CD21). Data were compared between two groups with a paired Student’s t test. *P <* 0.05 was considered statistically significant. ns, not significant; **P < 0.01; ***P < 0.001; ****P < 0.0001.

To characterize and fully understand the differentiation stage of CD11c^+^ B cells in the PB of GD patients, we examined the cell-surface expression of conventional B-cell antigens and chemokine receptors and the intracellular expression of T-bet. The gating strategy for the analysis of paired CD11c^+^ B cells and CD11c^-^ B cells in each sample is shown in **Supplementary Figure S6**, and example gating plots of CD11c^+^ B cells and CD11c^-^ B cells were generated as shown in **Figure 4A**. In GD patients, the frequency and mean fluorescence intensity (MFI) of the CD38^+^ B cells, CD27^+^ B cells, and CD138^+^ B-cell compartments were enriched in CD11c^+^ B cells compared to paired CD11c^-^ B cells (**Figures 4B, C, E**), suggesting that CD11c^+^ B cells represent a subset of antigen-experienced B cells. The MFI, but not the frequency of IgD, was higher in CD11c^+^ B cells than in paired CD11c^-^ B cells (**Figure 4D**). The percentage and MFI of T-bet^+^ B cells were particularly increased in CD11c^+^ B cells compared to paired CD11c^-^ B cells (**Figure 4F**). An unconventional pattern of chemokine receptor expression was found in CD11c^+^ B cells, with significantly increased expression of CXCR3 and reduced expression of CXCR5 (**Figures 4G, H**), which are involved in migration to sites of inflammation (38). In addition, the population of CD32b^+^ B cells and CD21^low^ B cells, which have been reported to expand as potential autoreactive B cells in various autoimmune diseases (39–42), was enriched in CD11c^+^ B cells compared to paired CD11c^-^ B cells (**Figures 4I, J**).

### CD11c^+^ B Cells Infiltrated the Thyroid of GD Patients With a Phenotype Similar to That of CD11c^+^ B Cells in the PB

**Figure 5:**
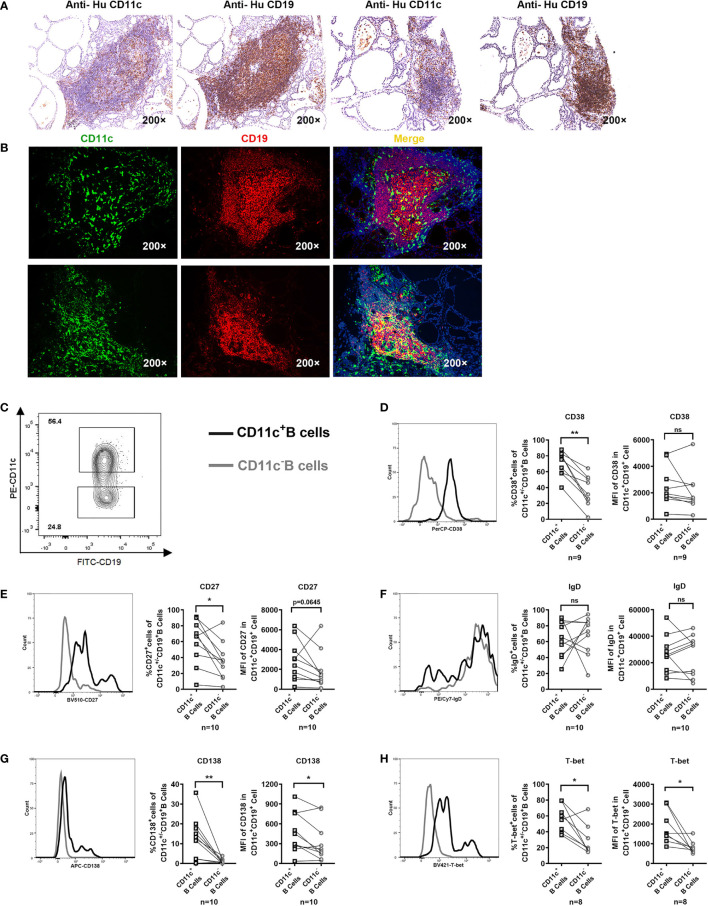
CD11c^+^ B cells in the thyroid of GD patients and phenotypic analysis. **(A)** Immunohistochemical staining for CD11c and CD19 in serial sections of thyroid tissue from two GD patients (200-fold magnification). **(B)** Immunofluorescence staining for CD11c (green) and CD19 (red) and DAPI (blue) in thyroid sections (200-fold magnification) from two GD patients. **(C)** Representative flow cytometric plots of paired CD11c^+^ B cells and CD11c^-^ B cells in FNA samples from GD patients. **(D–H)** Comparative overlapped histograms of FNA example in CD11c^+^B and CD11c^-^ B cells for compared markers (left). Comparison between the frequency of positive cell populations in CD11c^+^ B cells and that in paired CD11c^-^ B cells in FNA samples (middle). Comparison between the mean fluorescence intensities (MFIs) of the representative phenotype in CD11c^+^ B cells and those in paired CD11c^-^ B cells in FNA samples (right). The comparison metrics included conventional antigens (CD38, CD27, IgD, and CD138) and an unconventional antigen (T-bet). Data were compared between two groups with a paired Student’s t test. *P <* 0.05 was considered statistically significant. ns, no significance; *P < 0.05; **P < 0.01.

Immunostaining of serial thyroid sections showed that CD11c^+^ B cells were observed in the lymphocyte infiltration areas of the thyroid of GD patients (**Figure 5A**). The coexpression of CD11c (green) and CD19 (red) appeared in yellow (**Figure 5B**). Those CD11c-expressing cells that did not colocalize with CD19 may include dendritic cells, monocytes, macrophages, granulocytes, and natural killer cells (43, 44), which may play a complex role as antigen presenting cells and immune effector cells (45–47). Furthermore, we evaluated FNA samples by flow cytometry to further analyze the phenotype of CD11c^+^ B cells in the thyroid. The gating strategy for phenotypic analysis of paired CD11c^+^ B cells and CD11c^-^ B cells from FNA samples is shown in **Figure 5C**. The frequencies of CD38^+^, CD27^+^, and CD138^+^ cells and the MFI of CD138 were enriched in CD11c^+^ B cells from FNA samples compared to paired CD11c^-^ B cells (**Figures 5D, E, G**). Neither the positive cell proportion nor the MFI for IgD was found to be significantly different between paired CD11c^+^ B cells and CD11c^-^ B cells (**Figure 5F**). Both the positive cell frequency and MFI for T-bet were significantly increased in CD11c^+^ B cells compared to paired CD11c^-^ B cells (**Figure 5H**). The phenotypic characteristics of CD11c^+^ B cells in the FNA samples were consistent with those of CD11c^+^ B cells in the PB, which indicated that these cell populations might have the same origin.

### CD11c^+^ B Cells Further Differentiate in the Thyroid Compared With the PB in GD

**Figure 6:**
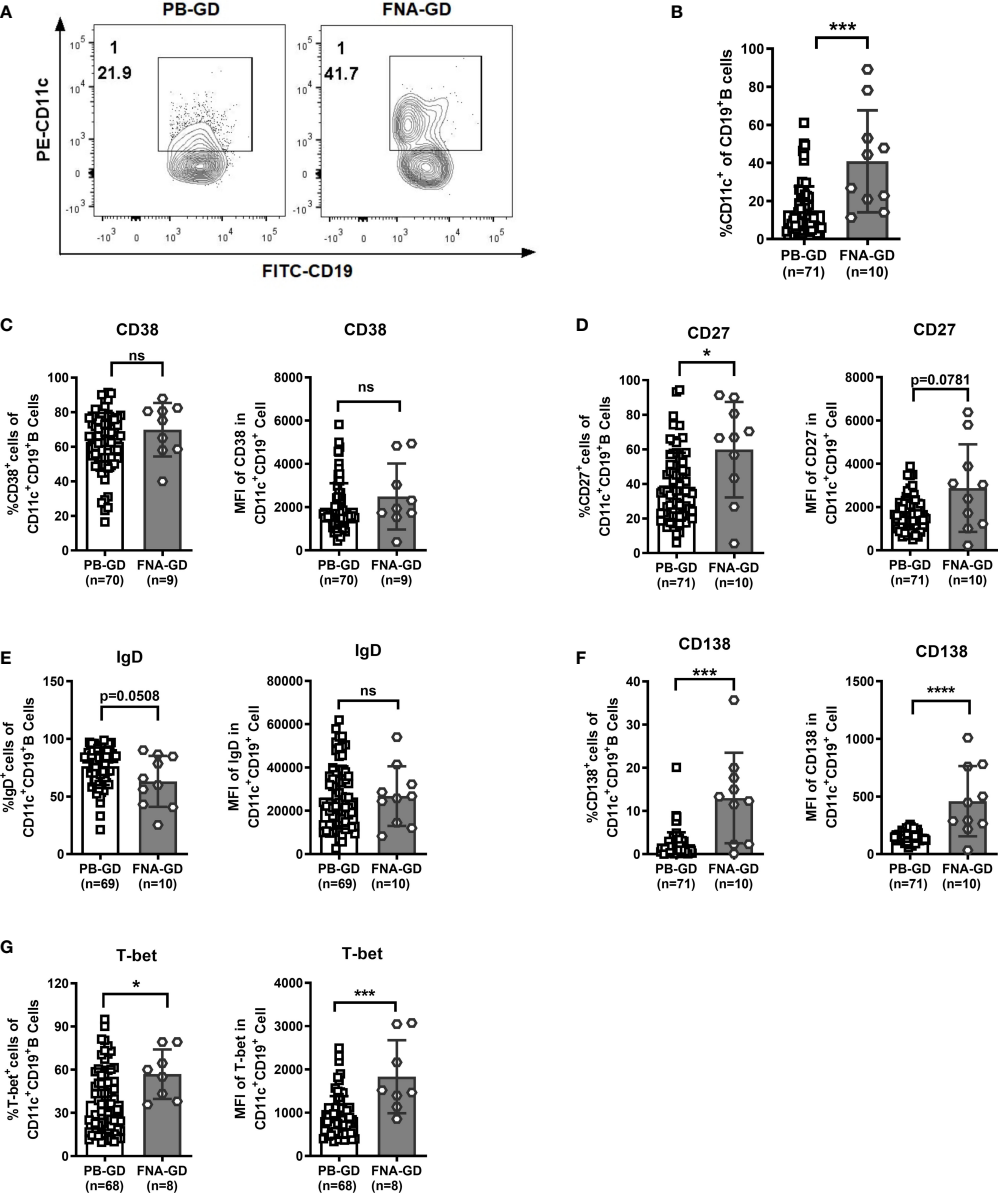
Comparison of CD11c^+^ B cells between peripheral blood (PB) and fine-needle aspiration (FNA) samples from GD patients. **(A)** Representative flow cytometric plots of CD11c^+^ B cells in PB and FNA samples from GD patients. **(B)** Frequency of CD11c^+^ B cells in CD19^+^ B cells for PB and FNA samples from GD patients. **(C–G)** Comparison between the frequency of the positive cell subset in PB CD11c^+^ B cells and that in FNA CD11c^+^ B cells (left). Comparison between the mean fluorescence intensities (MFIs) of the representative phenotype in PB CD11c^+^ B cells and that in FNA CD11c^+^ B cells (right). The comparison metrics included conventional antigens (CD38, CD27, IgD, and CD138) and an unconventional antigen (T-bet). Nonnormally distributed data are expressed as the median and IQR, assessed by nonparametric Mann–Whitney U tests. *P <* 0.05 was considered statistically significant. ns, not significant; *P < 0.05; ***P < 0.001; ****P < 0.0001.

We further compared the phenotype between circulating CD11c^+^ B cells in the PB and thyroid-infiltrated CD11c^+^ B cells in FNA samples by flow cytometry. The demographic and clinical characteristics of the enrolled GD patients are shown in **Table 2**. Both age (*P*=0.078) and sex (*P*=0.857) were well matched. No significant difference in patient thyroid autoantibody levels was observed between the PB group and FNA group. A higher frequency of CD11c^+^ B cells was observed in the FNA group than in the PB group (**Figure 6A, B**), which indicated that CD11c^+^ B cells accumulated in the thyroid compared to the PB. Next, we compared the phenotypes of CD11c^+^ B cells from PB samples and FNA samples and found that the percentages of CD27^+^ B cells and CD138^+^ B cells were significantly enriched in FNA samples, showing a higher MFI, compared to PB samples (**Figures 6D, F**). The frequency of IgD^+^CD11c^+^ B cells in FNA samples tended to show a decrease compared to that in the PB (**Figure 6E**), which indicated that CD11c^+^ B cells were postswitching and antigen experienced (19). In addition, CD11c^+^ B cells expressed T-bet at a higher frequency in the thyroid than in the PB (**Figure 6G**). The above findings indicated that CD11c^+^ B cells in the thyroid underwent further differentiation into ASCs compared to CD11c^+^ B cells in the PB.

**Table 2:** Demographic and clinical characteristics of GD patients in the peripheral blood and fine-needle aspiration (FNA) groups.

Population	Peripheral blood group	FNA group	p value
n	71	10	–
Sex M/F, n	16/55	2/8	0.857
Age (years)	50.11 (35.00-62.00)	35.00 (26.50-51.00)	0.078
TRAb (IU/ml)	7.34 (2.59-14.96)	3.39 (1.60-15.19)	0.438
TPOAb (IU/ml)	108.10 (23.24-278.1)	147.10 (96.72-418.10)	0.212
TgAb (IU/ml)	107.30 (15.48-483.10)	299.10 (19.45-557.40)	0.963
CD11c^+^B cells (% in total B cells)	11.20 (5.79-20.10)	35.60 (19.28-59.30)	<0.001

TRAb, anti-thyrotropin receptor antibody; TgAb, anti-thyroglobulin antibody; TPOAb, anti-thyroid peroxidase antibody.

The thyroid autoantibodies in the two groups of participants are presented as serum concentrations.

Nonnormally distributed data are expressed as the median and IQR. The counting data were analyzed by a chi-square test. Continuous variables with nonnormal distributions were assessed with Mann–Whitney U tests. P < 0.05 was considered statistically significant.

### CD11c^+^ B Cells are Poised to Differentiate to Plasma Cells and Produce TRAbs *In Vitro*

**Figure 7:**
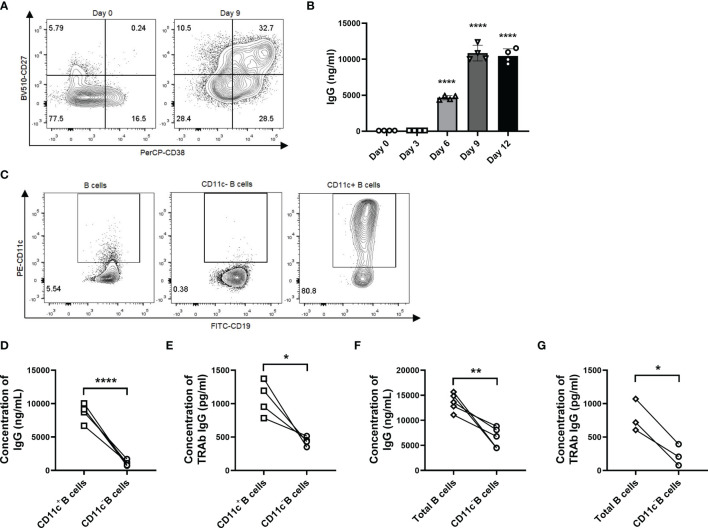
CD11c^+^ B cells are activated to differentiate into autoreactive ASCs that secrete IgG and TRAbs. **(A)** Representative flow cytometric plots of plasmablasts (CD27^high^CD38^high^ B cells, top right panel) in CD19^+^ B cells from GD patients before culture (left, day 0) and after 9 days of culture (right, day 9). **(B)** The measurement of IgG in culture supernatants was carried out continuously for up to 12 days. Data were assessed by ANOVA, and the groups were compared between day 0 and days 3, 6, 9, and 12, with the results marked above the histogram bars. **(C)** Representative flow cytometric plots of total CD19^+^ B cells, CD11c^-^ B cells, and CD11c^+^ B cells from GD patients. **(D)** Comparison of the IgG concentration in culture supernatants between paired CD11c^+^ B cells and CD11c^-^ B cells in each batch from different GD patients. **(E)** Comparison of the TRAb concentration in culture supernatants between paired CD11c^+^ B cells and CD11c^-^ B cells in each batch from different GD patients. **(F)** Comparison of the IgG concentration in culture supernatants between paired total B cells and CD11c^-^ B cells in each batch from different GD patients. **(G)** Comparison of the TRAb concentration in culture supernatants between paired total B cells and CD11c^-^ B cells in each batch from different GD patients. Data are represented as the mean ± SD and were compared between two groups with a paired Student’s t test. *P <* 0.05 was considered statistically significant. ns, not significant; *P < 0.05; **P < 0.01; ****P < 0.0001.

As our evidence suggested that CD11c^+^ B cells showed a strong correlation with TRAb levels and that their phenotype was associated with ASCs, we sorted and induced CD11c^+^ B cells *in vitro* to clarify whether these cells could differentiate into autoreactive ASCs that produce autoantibodies. We found that TLR7 agonist-induced total B cells from GD patients differentiated into ASCs and that IgG in culture supernatants was strikingly increased on day 9 (**Figures 7A, B** and **Supplementary Figure S7**). Next, we applied the culture system to separately evaluate total B cells, CD11c^-^B cells, and CD11c^+^ B cells from GD patients (**Figure 7C**). We found that the concentrations of both IgG and TRAbs were significantly increased in CD11c^+^ B cells compared to paired CD11c^-^ B cells (**Figures 7D, E**). Depletion of CD11c^+^ B cells from the total B-cell population substantially attenuated the secretion of IgG and TRAbs (**Figures 7F, G**).

### Cytokine Secretion Profile of CD11c^+^ B Cells From GD Patients

**Figure 8:**
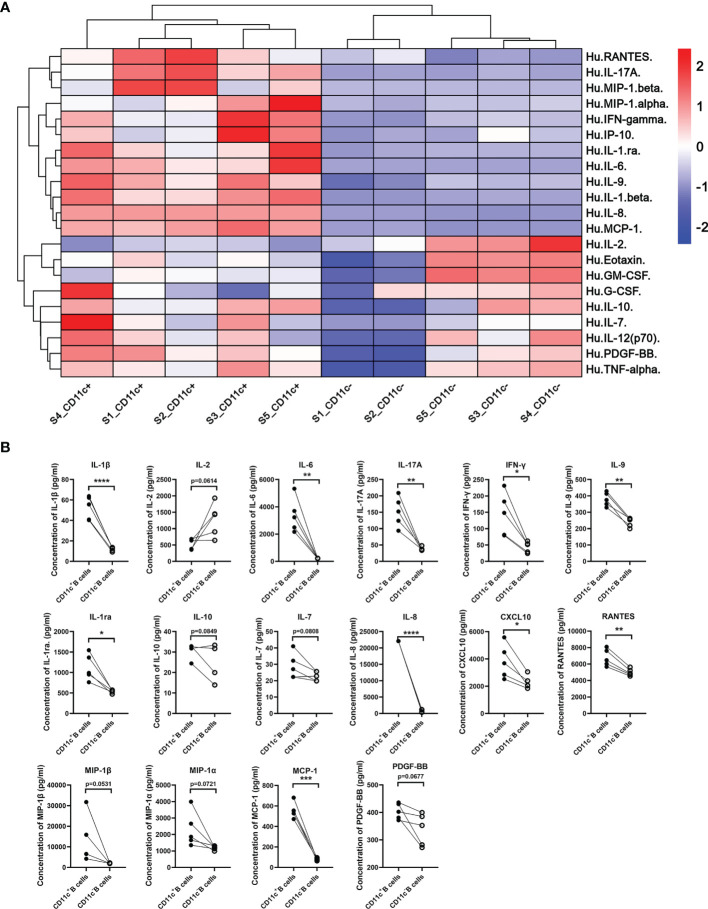
Cytokine secretion profile of paired CD11c^+^ B cells and CD11c^-^ B cells from GD patients. **(A)** Cytokine secretion pattern heatmap of paired CD11c^+^ B cells and CD11c^-^ B cells from 5 batches of GD patients evaluated with a Luminex liquid suspension chip. **(B)** Cytokines with a significant change or notable trend between paired CD11c^+^ B cells and CD11c^-^ B cells are displayed. Paired Student’s t test. *P <* 0.1 was considered indicative of a trend; *P <* 0.05 was considered statistically significant. ns, not significant; *P < 0.05; **P < 0.01; ***P < 0.001; ****P < 0.0001.

To further elucidate the cytokine secretion pattern of CD11c^+^ B cells from GD patients, we detected the cytokines in culture supernatants secreted by PB CD11c^+^ B cells, including a variety of interleukins, interferons, chemokines, and growth factors. The cytokine concentration of all samples was detected above the lower limit of detection (LLOD) in a Luminex liquid suspension chip assay. Our data showed that CD11c^+^ B cells exhibited a cytokine profile distinct from that of paired CD11c^-^ B cells. All detectable cytokines are shown as a heatmap in **Figure 8A**. The levels of proinflammatory cytokines, including IL-1β, IL-6, IL-17A, IFN-γ, and IL-9, were significantly higher in CD11c^+^ B cells than in CD11c^-^ B cells (all *P*<0.05, **Figure 8B**). The levels of anti-inflammatory cytokines (IL-1ra *P*<0.05, IL-10 *P*=0.085, **Figure 8B**) were also increased in the supernatant of CD11c^+^ B cells, indicating that these factors may exert a protective effect to limit immune responses. A number of chemokines, including IL-8 (CXCL8), CXCL10, RANTES (CCL5), MIP-1α/β, and monocyte chemoattractant protein-1 (MCP-1, CCL2), were significantly increased in CD11c^+^ B cells compared to paired CD11c^-^ B cells after activation with PHA. These results indicated that CD11c^+^ B cells might be involved in the migration of various immune cells to inflammatory sites in the thyroid. The concentration of platelet-derived growth factor-BB (PDGF-BB) was increased in the supernatant of CD11c^+^ B cells compared to that of paired CD11c^-^ B cells, which was reported to exacerbate the immunopathological responses of orbital fibroblasts in GO (48). The above findings for multiple cytokines indicated that CD11c^+^ B cells might play a complex role in the development of GD.

### CD11c^+^ B Cells Were Recruited by TFCs *via* the CXCR3-CXCL10 Axis

**Figure 9:**
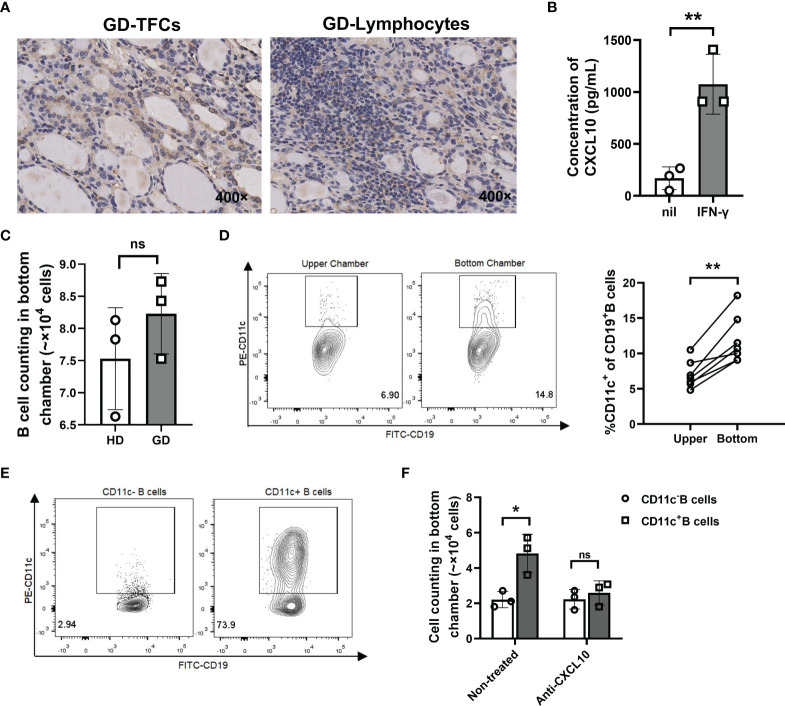
CD11c^+^ B cells were recruited to the thyroid *via* the CXCR3-CXCL10 axis. **(A)** Immunohistochemical staining for CXCL10 in thyroid sections from GD patients (400-fold magnification). **(B)** Concentration of CXCL10 in culture supernatants of Nthy-ori3-1 cells under control conditions or stimulation with IFN-γ (1000 U/ml) for 72 h. **(C)** Transwell assay performed with total CD19^+^ B cells from healthy donors (control) and GD patients. After 3 hours, the cells that migrated to the lower chamber were counted. **(D)** Representative flow cytometric plots of CD11c^+^ B cells from the paired upper chamber and lower chamber after 3 h of migration (left). Comparison of the frequency of CD11c^+^ B cells in CD19^+^ B cells between the paired upper chamber and lower chamber (right). **(E)** Flow cytometric plots of sorted CD11c^-^ B cells (left) and CD11c^+^ B cells (right) from GD patients. **(F)** Transwell assays performed with CD11c^-^ B cells and CD11c^+^ B cells from GD patients. Untreated and anti-CXCL10 antibody-treated culture supernatants were added to the lower chamber separately. After 3 h, the cells that migrated to the lower chamber were counted. Data are represented as the mean ± SD, paired or unpaired Student’s t test. *P <* 0.05 was considered statistically significant. ns, not significant; *P < 0.05; **P < 0.01.

It has been reported that IFN-γ and CXCL10 are increased in both the thyroid tissue and circulation of recent-onset GD patients and play critical roles in recruiting CXCR3^+^ lymphocytes into the thyroid (49–51). Thus, we speculated that CXCL10 may recruit CD11c^+^ B cells by binding to CXCR3. We first confirmed the presence of CXCL10 in both TFCs and lymphocyte infiltration areas of GD thyroid tissue (**Figure 9A**). A thyroid cell line (Nthy-ori3-1) secreted CXCL10 after stimulation with IFN-γ (**Figure 9B**). Furthermore, we used a Transwell chamber to determine the migratory capacity of CD11c^+^ B cells. Culture supernatants of Nthy-ori3-1 stimulated with IFN-γ (referred to as “IFN-γ treated supernatants”) were placed in the bottom chamber to mimic the microenvironment of the recent-onset GD thyroid. We found that the number of migrated B cells showed no significant difference between GD patients and HD (**Figure 9C**), but the frequency of CD11c^+^ B cells in the bottom chamber was significantly increased compared to that in the upper chamber (**Figure 9D**), which indicated that CD11c^+^ B cells showed a higher migratory capacity than other CD19^+^ B cells. Next, we loaded sorted CD11c^+^ B cells and CD11c^-^ B cells from GD patients into the upper chambers, and IFN-γ-treated supernatants were added to the bottom chamber (**Figure 9E**). After 3 h, we found that more CD11c^+^ B cells than CD11c^-^ B cells migrated, and inhibition with an anti-CXCL10 antibody significantly reduced the percentage of migrated CD11c^+^ B cells in the bottom chamber (**Figure 9F**). The above findings suggested that CD11c^+^ B cells were recruited by TFCs *via* the CXCR3-CXCL10 axis.

## Discussion

**Figure 10:**
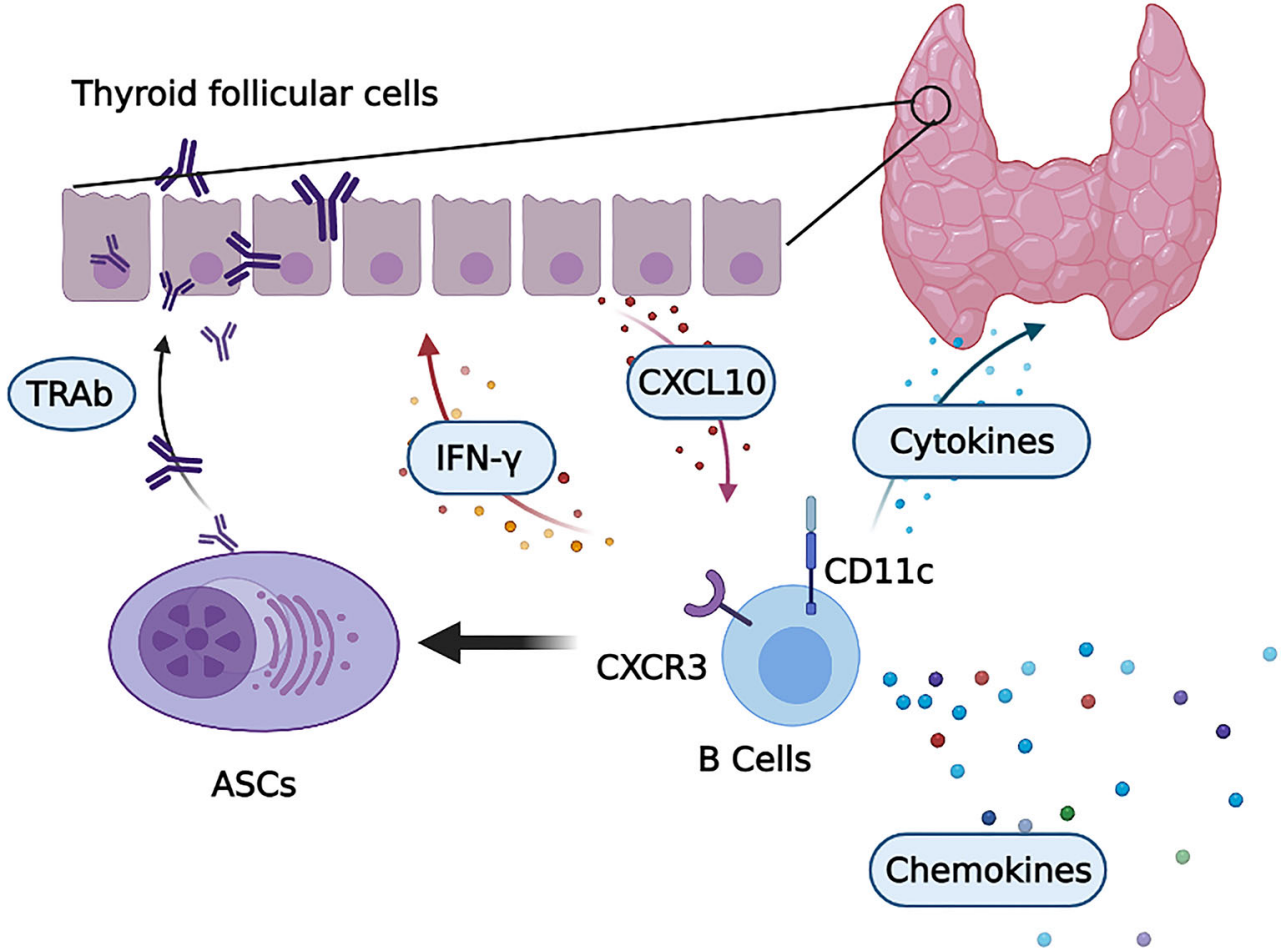
CD11c^+^ B cells in the thyroid are involved in the pathogenesis of GD. Infiltrated CD11c^+^ B cells with high CXCR3 expression were induced to differentiate into autoreactive antibody-secreting cells (ASCs) that secrete thyroid autoantibodies, including TRAbs. Activated CD11c^+^ B cells secrete a variety of cytokines, such as IFN-γ, to exacerbate local thyroid inflammation. This process induced thyroid follicular cells (TFCs) to secrete CXCL10, which recruited CXCR3-expressing lymphocytes, including CD11c^+^ B cells. Then, CD11c^+^ B cells and TFCs formed a positive inflammatory feedback loop. In addition, a number of chemokines were secreted by CD11c^+^ B cells, which potentiated lymphocyte infiltration into the thyroid of GD patients.

Here, we identified a unique B-cell subset expressing CD11c that expanded in the PB and accumulated in the thyroid of GD patients. We found that CD11c^+^ B cells play an important role in the pathogenesis of GD in the thyroid, as shown in **Figure 10**. Distinct expression patterns of chemokine receptors were observed in CD11c^+^ B cells, with increased expression of CXCR3 and downregulation of CXCR5, which facilitated the recruitment of these cells to sites of inflammation (49). Consistent with previous studies (52, 53), our study proved that TFCs secreted CXCL10 after stimulation with IFN-γ, which recruited CD11c^+^ B cells by binding to CXCR3. CD11c^+^ B cells secreted IFN-γ after activation, which indicated that infiltrated CXCR3^+^CD11c^+^ B cells and TFCs might form a positive feedback loop to recruit CXCR3^+^ lymphocytes to infiltrate the thyroid gland (53). CD11c^+^ B cells showed intrinsically high expression of T-bet, which is essential for the IFN-γ-induced differentiation of ASCs (54), and were poised to differentiate into autoreactive ASCs that secrete IgG and TRAbs. In addition, the unique pattern of chemokines indicated that CD11c^+^ B cells played a critical role in recruiting various lymphocytes into the thyroid, thereby expanding the area of lymphocyte infiltration and promoting the occurrence and development of GD. We first demonstrated that CD11c^+^ B cells, as an autoreactive and pathogenic B-cell subset, are involved in the pathogenesis of GD in multiple ways.

Single-cell sequencing of SLE and primary Sjögren’s syndrome patients in a previous study showed that CD11c^+^ B cells were enriched in atypical memory cells (17). In our study, phenotypic analysis showed that CD11c^+^ B cells in GD were associated with the populations of memory cells, plasmablasts, and plasma cells. Remarkably, the frequency of CD11c^+^ B cells was positively correlated with the titers of thyroid autoantibodies, especially TRAbs, which indicated that CD11c^+^ B cells might participate in the onset of GD by secreting thyroid autoantibodies. Consistent with previous studies in other autoimmune diseases (17), some inhibitory expression patterns, such as the CD21^lo^CD32b^hi^CD11c^+^ phenotype, were also observed in the CD11c^+^ B cells of GD patients. CD21^lo^ B cells were reported to represent anergic B cells (55) and to be enriched in a number of autoimmune diseases. CD32b, an inhibitory receptor, was observed to suppress the activation of autoreactive B cells with defective central tolerance and restrict ASC differentiation (56, 57). The inhibitory expression patterns of CD11c^+^ B cells may act as self-limited regulatory surface factors of these autoreactive B cells to maintain a relatively low frequency in the periphery of HD.

Our data indicated that CD11c^+^ B cells played a critical role in the production of autoantibodies in GD. In a previous study, IgH repertoire analysis indicated that in CD11c^+^ B cells from SLE patients, a defect in negative selection during GC transit with overexpression of V4-34 was present, which was associated with autoimmune diseases (18, 58, 59). The measurement of somatic mutation frequencies in CD11c^+^ B cells from SLE showed the mutational frequencies typical of a GC experience (18). We found that CD11c^+^ B cells showed a greater capacity for IgG secretion than CD11c^-^ B cells, including but not exclusive to TRAb. A variety of autoantibodies were detected to be significantly correlated with CD11c^+^ B cells in SLE (19). The above findings indicate that CD11c^+^ B cells that escape central immune tolerance (60, 61) may lead to the secretion of a wide range of autoantibodies, including TRAbs, which may be an indicator of individuals with a high risk of developing other autoimmune diseases.

In addition to the function of autoantibody secretion, CD11c^+^ B cells also participate in the pathogenesis of GD in multiple ways by secreting a variety of cytokines, exhibiting significantly increased secretion of proinflammatory cytokines (IL-1β, IL-6, IL-17A, IFN-γ, and IL-9), anti-inflammatory cytokines (IL-1ra and IL-10), and chemokines (IL-8, CXCL10, RANTES, MIP-1α/β, and MCP-1). Marie-Laure Golinski and colleagues (62) reported that the elevated cytokines in CD11c^+^ B cells from HD partly overlapped with our results in GD patients, including the results for IFN-γ, IL-1β, and IL-10. The above findings indicated the heterogeneous cytokine expression profiles of CD11c^+^ B cells among different diseases and populations. In addition, Kemp EH and colleagues (63) reported that the expression of various chemokines was significantly increased in thyroid tissues from patients with autoimmune thyroid diseases (AITD) compared to those from non-AITD patients, which highly coincided with the chemokine secretory pattern of CD11c^+^ B cells observed in our study. This observation indicates that CD11c^+^ B cells may play a critical role in recruiting inflammatory cells into the thyroid and exacerbating local lymphocyte infiltration. We speculated that CD11c^+^ B cells with heterogeneous cytokine profiles have complex functions and are involved in multiple biological processes to promote the development of GD.

Previous studies reported that the accumulation of CD11c^+^ B cells was driven by the activation of TLR7 (64–66). In the current study, after activation of TLR7 *in vitro*, CD11c^+^ B cells from GD patients were induced to differentiate into ASCs capable of secreting IgG and TRAbs in the microenvironment of GD. A higher intensity of intracellular TLR7 was observed in GD patients, especially intractable GD patients, than in HD (67), which may play a critical role in regulating the extrafollicular B-cell response, leading to the expansion of self-reactive CD11c^+^ B cells to breakdown B-cell homeostasis (27). Nagata, K. and colleagues (68) reported that Epstein–Barr virus (EBV) reactivation was associated with TRAb production in GD. EBV noncoding RNAs are recognized by TLR7, a sensor of single-stranded RNA of B cells (69). EBV infection may be involved in the differentiation of autoreactive B cells and autoantibody secretion by activating TLR7. The above findings indicated that both genetic factors and environmental factors, including infection, have been proposed as risk factors for Graves’ disease.

The present study had some limitations. First, our study investigated only CD11c^+^ B cells in GD and described the multiple functions of this unique B-cell subset in GD. Since the proportions of CD11c^+^ B cells in most HD were relatively low, it was difficult to further isolate and investigate the function of CD11c^+^ B cells in HD. Secondly, we did not obtain PB and FNA samples from the same GD patient, but the participants in these two groups were successfully matched for age, sex and titers of thyroid autoantibodies. Last, we did not evaluate the changes in CD11c^+^ B cells in a single individual before and after ATD treatment. A larger population and long-term follow-up are essential for elucidating the dynamic relationship between CD11c^+^ B cells and TRAbs to illuminate the critical role of CD11c^+^ B cells in the pathogenesis of GD.

In conclusion, we reported that autoreactive CD11c^+^ B cells were expanded in GD patients and involved in the pathogenesis of GD by secreting thyroid autoantibodies and a variety of cytokines, which might exacerbate local inflammation and lymphocyte infiltration in thyroid tissue. The current management of GD, including ATD, RAI, and thyroidectomy (9, 21), either shows limited efficacy in many patients or results in lifelong thyroid hormone replacement therapy. The demand for novel therapeutic options has led to the emergence of immunotherapeutic approaches targeting B cells in GD patients (70, 71). Therefore, the CD11c^+^ B-cell subset may be a promising immunotherapeutic target in the future.

## Data Availability Statement

The original datasets analyzed in the current study are available from the corresponding author on reasonable request.

## Ethics Statement

The studies involving human participants were reviewed and approved by Ethics Committee of Peking University First Hospital. The patients/participants provided their written informed consent to participate in this study.

## Author Contributions

The concept was conceived by YC, YGa, and JZ. The overall study design was developed by YC and YGa. The experiments were performed by YC, XZ, RY, YGo and CQ. The data analysis was performed by YC, YZ, and CQ. The sample resources were provided by YH, YY, YZ, YGo, TC, EZ, and LZ. The manuscript was written by YC and YGa. The research was supervised by YGao. All authors contributed to the article and approved the submitted version.

## Funding

This work was supported by the National Natural Science Foundation of China [grant numbers 81770783 and 82170801].

## Conflict of Interest

The authors declare that the research was conducted in the absence of any commercial or financial relationships that could be construed as a potential conflict of interest.

## Publisher’s Note

All claims expressed in this article are solely those of the authors and do not necessarily represent those of their affiliated organizations, or those of the publisher, the editors and the reviewers. Any product that may be evaluated in this article, or claim that may be made by its manufacturer, is not guaranteed or endorsed by the publisher.

## Supplementary Material

Supplementary Figure S1 Gating strategy of CD19^+^ B cells and CD11c^+^ B cells. **(A)** Single cells were plated for further analysis. **(B)** Lymphocytes are circled in the single-cell gate. **(C)** B cells are circled as CD19^+^ cells in the lymphocyte gate. **(D)** CD11c^+^ B cells are circled in the CD19^+^ B-cell gate.

Supplementary Figure S2 Frequency of CD19^+^ B cells in HD and GD patients. Frequency of CD19^+^ B cells in peripheral blood mononuclear cells (PBMCs) compared between HD and GD patients. Nonnormally distributed data are expressed as the median and IQR, assessed by Mann–Whitney U tests. *P <*0.05 was considered statistically significant. ns, not significant.

Supplementary Figure S3 Analysis of the correlations between the frequency of CD11c^+^ B cells and age in GD patients. Linear correlation analysis between age and the frequency of CD11c^+^ B cells in CD19^+^ B cells for all enrolled GD patients. The correlation analysis was performed using the Spearman correlation test. *P <* 0.05 was considered statistically significant.

Supplementary Figure S4 Analysis of the correlations between the frequency of CD11c^+^ B cells and thyroid function. **(A–C)** Linear correlation analysis between the frequency of CD11c^+^ B cells in CD19^+^ B cells and thyroid function markers (TSH, fT4, and fT3) in all enrolled GD patients. The correlation analyses above were performed using the Spearman correlation test.

Supplementary Figure S5 Global gating strategy for analysis of CD11c expression distribution. After gating the single cells and lymphocytes, B cells were circled as CD19^+^ cells for further analysis. Then, B cells were divided into 13 subsets according to the expression of IgD, CD27, CD38, and CD138. The B-cell subsets are as follows: Q1-naïve B cells (CD27^-^IgD^+^), Q2-unswitched memory B cells (CD27^+^IgD^+^), Q3-switched memory B cells (CD27^+^IgD^-^), Q4-double negative memory B cells (CD27^-^IgD^-^), Q5-naïve mature B cells (CD38^-^IgD^+^), Q6-activated naïve mature B cells (CD38^+^IgD^+^), Q7-early memory mature B cells/germinal center B cells (CD38^+^IgD^-^/CD38^high^IgD^-^), Q8-resting memory B cells (CD38^-^IgD^-^), Q9-transitional B cells (CD38^-^CD27^+^), Q10-plasmablasts (CD38^+^CD27^+^), Q11-transitional-like B cells (CD38^+^CD27^-^), Q12-memory B-cell precursors (CD38^-^CD27^-^), and plasma cells (CD138^+^). CD11c^+^ B cells were circled in the above B-cell subsets.

Supplementary Figure S6 Global gating strategy for comparing immune marker expression between CD11c^+^ and CD11c^-^ B cells. CD19^+^ B cells were gated for the following analysis, and CD11c^+/high^ and CD11c^-^ B cells were circled to analyze the frequency and MFI of the positive subpopulation among all immunomarkers, including CD27, CD38, IgD, CD138, T-bet, CXCR5, CXCR3, CD32, and CD21.

Supplementary Figure S7 IgG production of B cells stimulated with different concentrations of R848. Total B cells from GD patients were stimulated with a concentration gradient of R848 (a TLR7/8 agonist). The culture supernatants were collected on day 9 and measured by ELISA. Data are presented as the mean ± SD and were assessed by ANOVA. The 0.1, 1, and 10 μg/ml groups were compared with the 0 μg/ml group, and the results are marked above the histogram bars. *P <* 0.05 was considered statistically significant. ns, not significant; *****P <* 0.0001.

Supplementary Table S1 Antibodies used in flow cytometry analysis.

Supplementary Table S2 Antibodies used in multiplex immunofluorescence staining.

Supplementary Table S3 The lower limits of detection (LLOD) of all cytokines in the Luminex liquid suspension chip.
