# Supplementary figures and images for: CD11c+ B Cells Participate in the Pathogenesis of Graves’ Disease by Secreting Thyroid Autoantibodies and Cytokines
Source: Front Immunol. 2022 Mar 21;13:836347. doi: 10.3389/fimmu.2022.836347 (PMC8977450; doi:10.3389/fimmu.2022.836347)

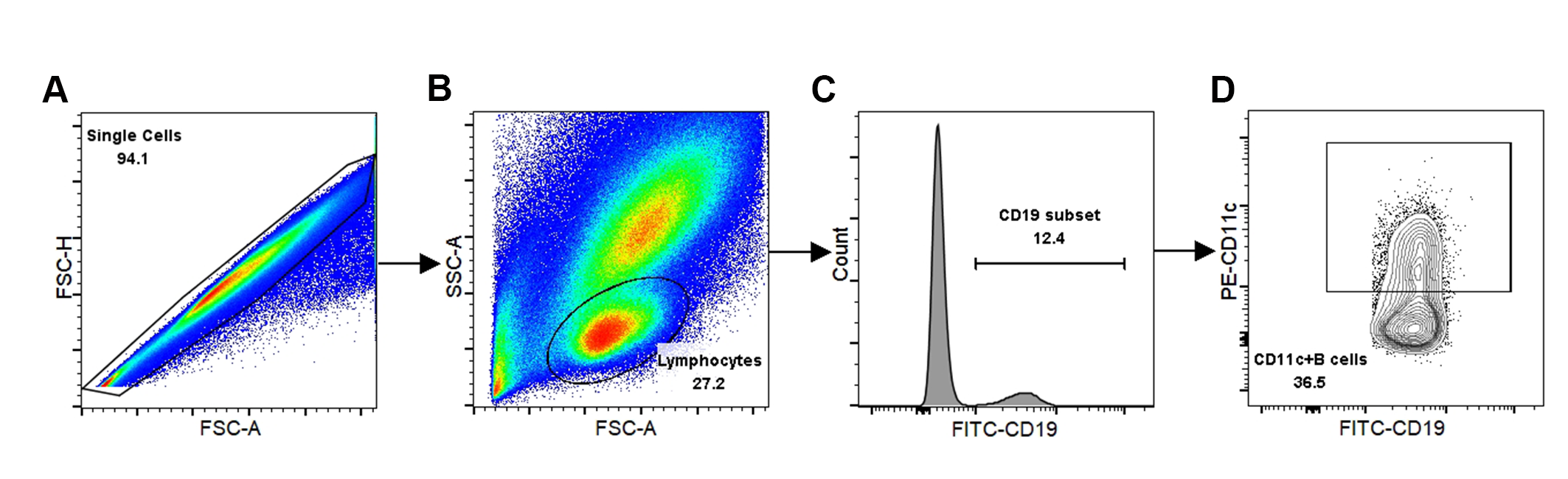

Supplement: Supplementary Figure S1 — Gating strategy of CD19+ B cells and CD11c+ B cells. (A) Single cells were plated for further analysis. (B) Lymphocytes are circled in the single-cell gate. (C) B cells are circled as CD19+ cells in the lymphocyte gate. (D) CD11c+ B cells are circled in the CD19+ B-cell gate. [file Image_1.tif]

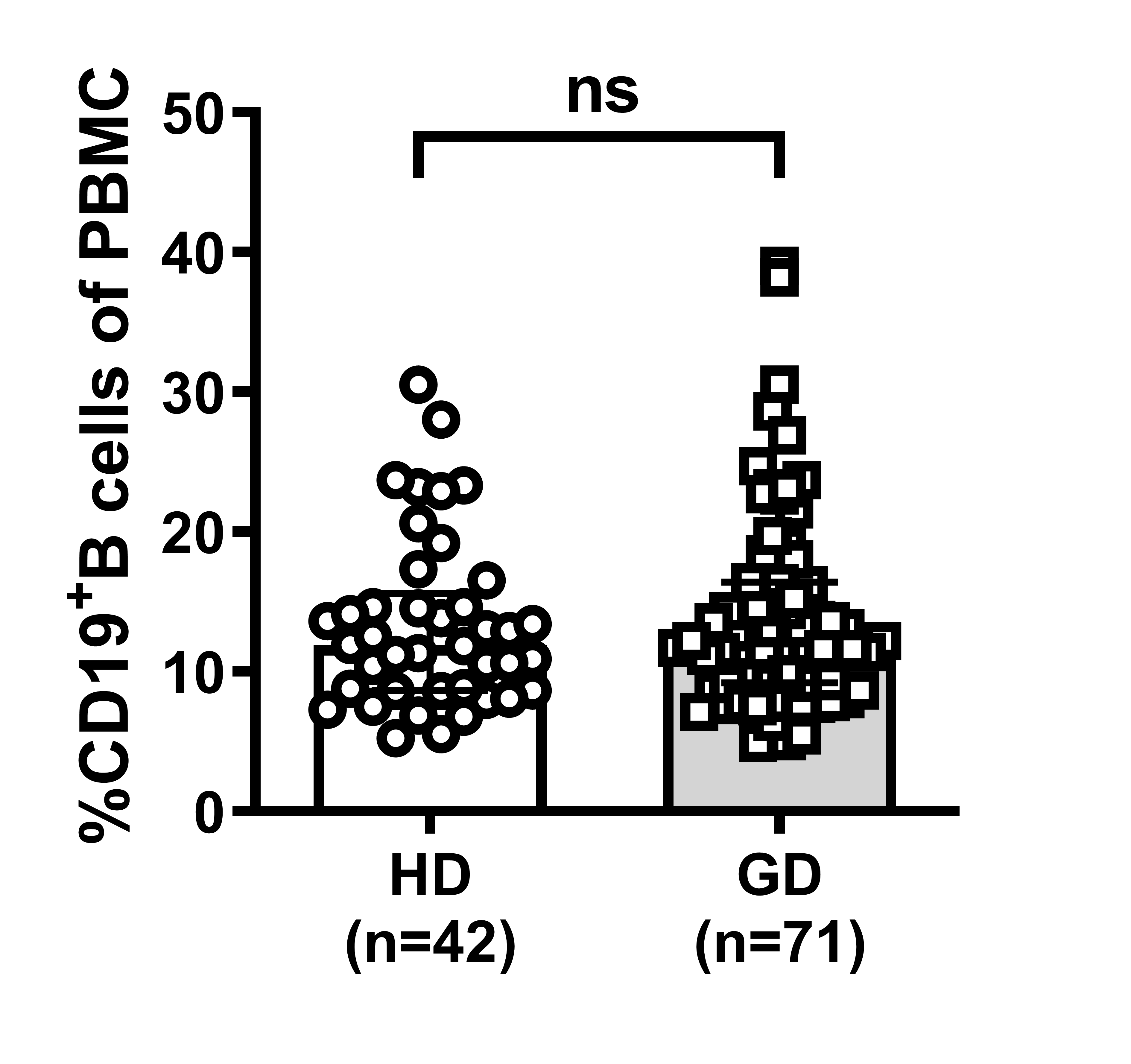

Supplement: Supplementary Figure S2 — Frequency of CD19+ B cells in HD and GD patients. Frequency of CD19+ B cells in peripheral blood mononuclear cells (PBMCs) compared between HD and GD patients. Nonnormally distributed data are expressed as the median and IQR, assessed by Mann–Whitney U tests. P <0.05 was considered statistically significant. ns, not significant. [file Image_2.tif]

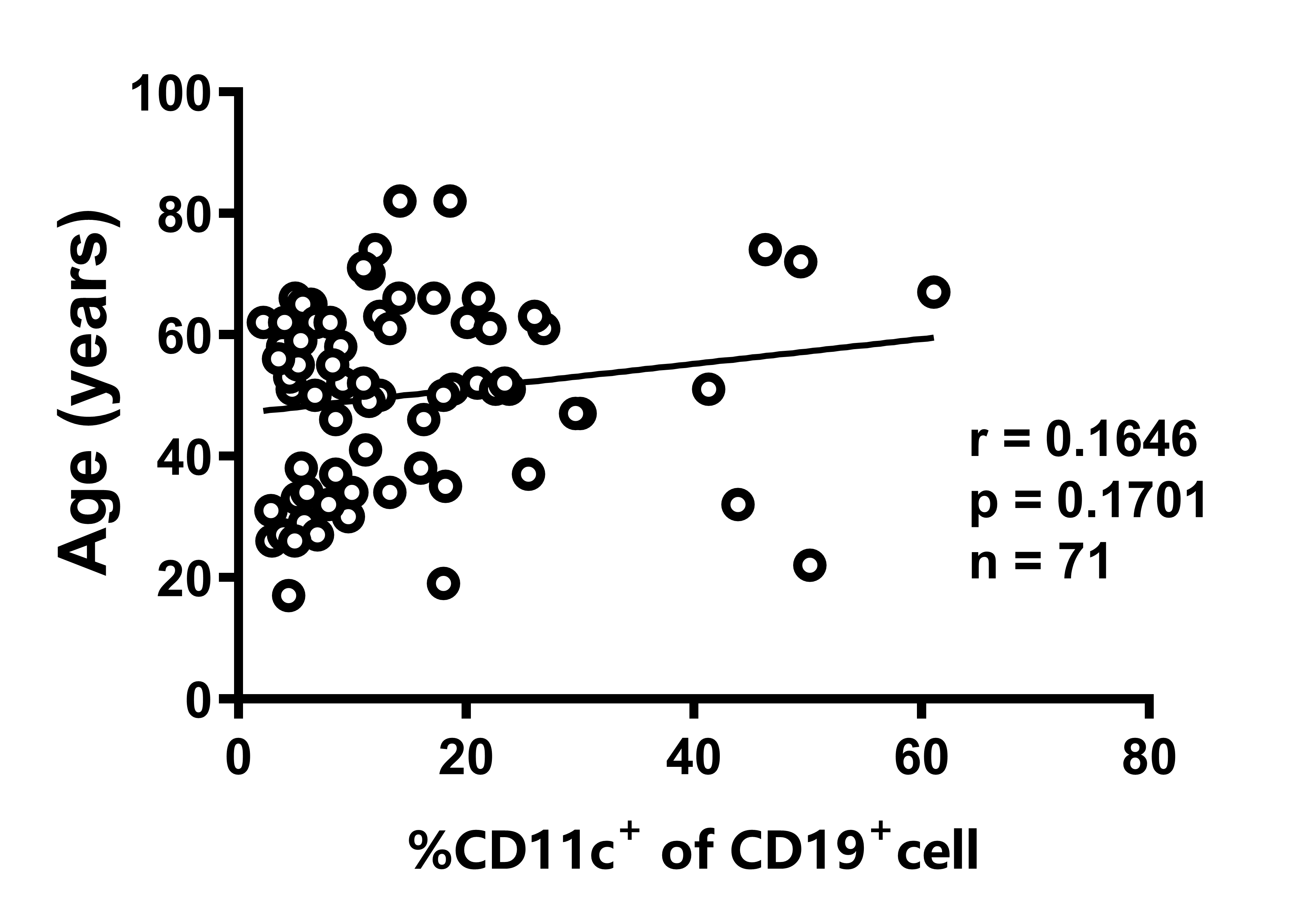

Supplement: Supplementary Figure S3 — Analysis of the correlations between the frequency of CD11c+ B cells and age in GD patients. Linear correlation analysis between age and the frequency of CD11c+ B cells in CD19+ B cells for all enrolled GD patients. The correlation analysis was performed using the Spearman correlation test. P < 0.05 was considered statistically significant. [file Image_3.tif]

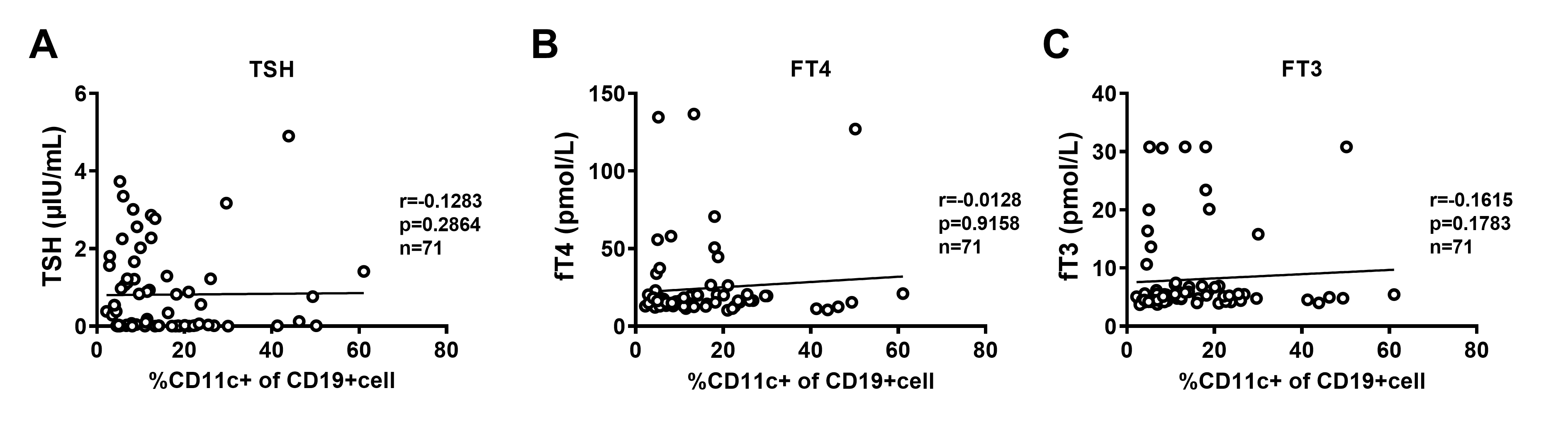

Supplement: Supplementary Figure S4 — Analysis of the correlations between the frequency of CD11c+ B cells and thyroid function. (A–C) Linear correlation analysis between the frequency of CD11c+ B cells in CD19+ B cells and thyroid function markers (TSH, fT4, and fT3) in all enrolled GD patients. The correlation analyses above were performed using the Spearman correlation test. [file Image_4.tif]

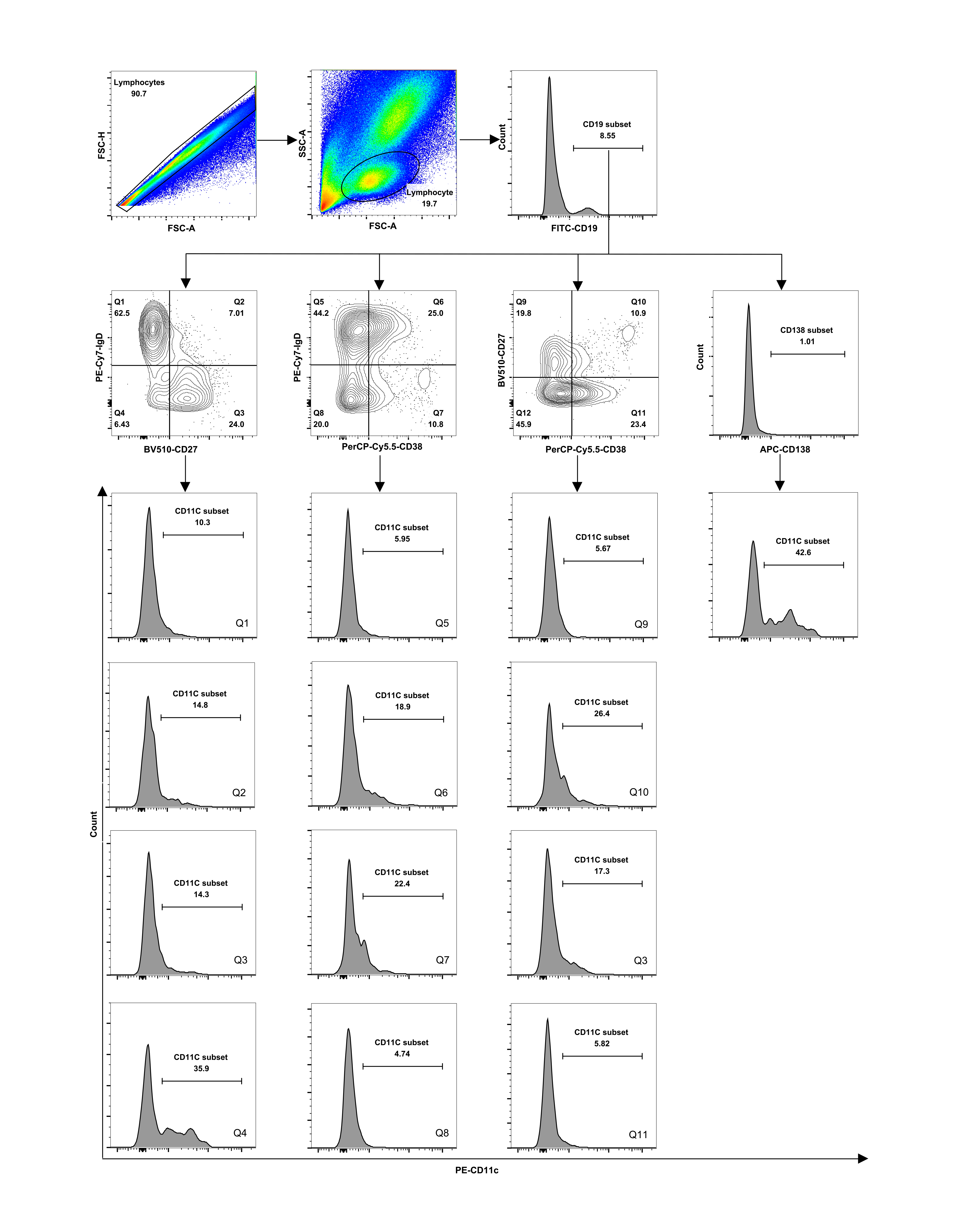

Supplement: Supplementary Figure S5 — Global gating strategy for analysis of CD11c expression distribution. After gating the single cells and lymphocytes, B cells were circled as CD19+ cells for further analysis. Then, B cells were divided into 13 subsets according to the expression of IgD, CD27, CD38, and CD138. The B-cell subsets are as follows: Q1-naïve B cells (CD27-IgD+), Q2-unswitched memory B cells (CD27+IgD+), Q3-switched memory B cells (CD27+IgD-), Q4-double negative memory B cells (CD27-IgD-), Q5-naïve mature B cells (CD38-IgD+), Q6-activated naïve mature B cells (CD38+IgD+), Q7-early memory mature B cells/germinal center B cells (CD38+IgD-/CD38highIgD-), Q8-resting memory B cells (CD38-IgD-), Q9-transitional B cells (CD38-CD27+), Q10-plasmablasts (CD38+CD27+), Q11-transitional-like B cells (CD38+CD27-), Q12-memory B-cell precursors (CD38-CD27-), and plasma cells (CD138+). CD11c+ B cells were circled in the above B-cell subsets. [file Image_5.tif]

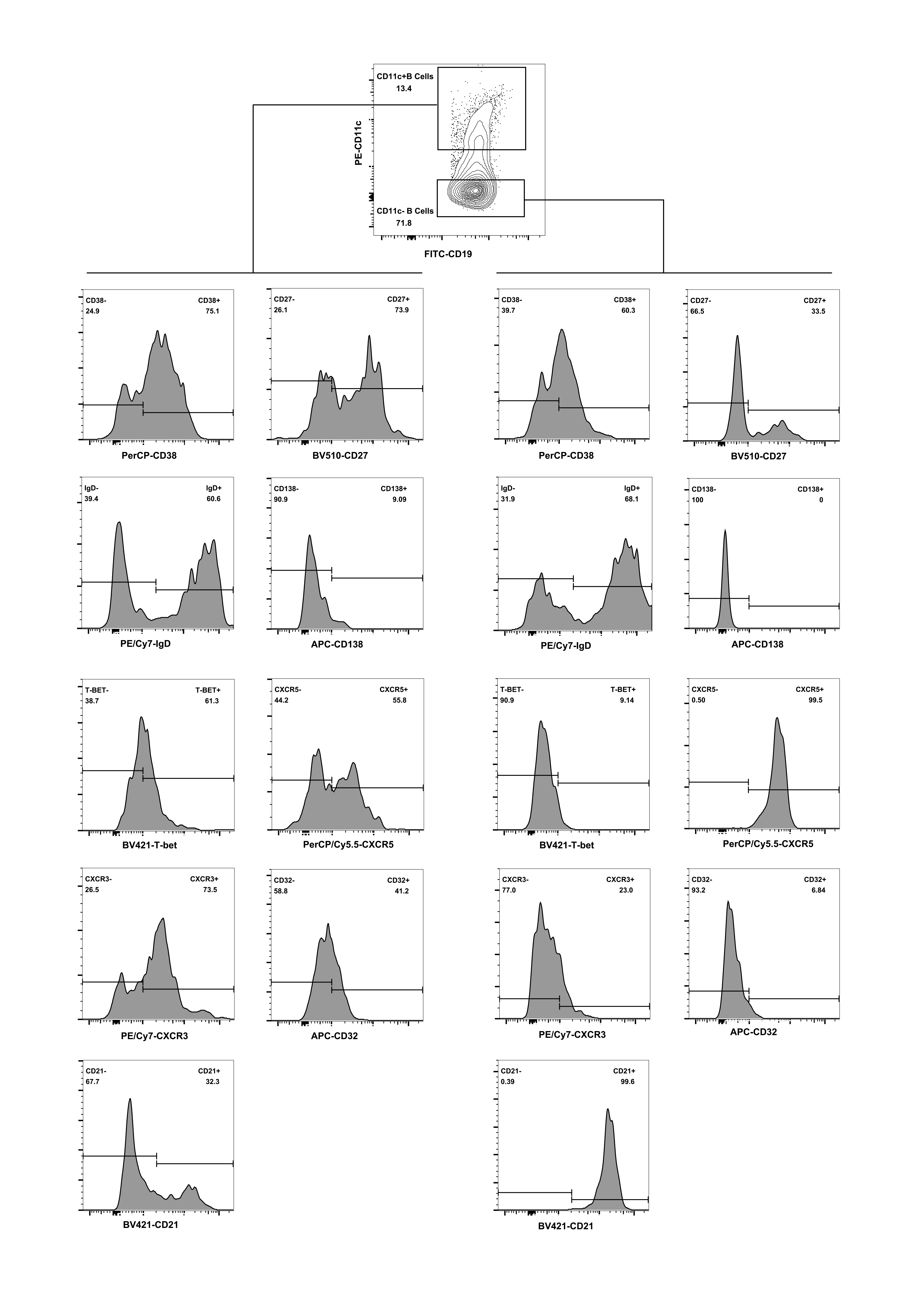

Supplement: Supplementary Figure S6 — Global gating strategy for comparing immune marker expression between CD11c+ and CD11c- B cells. CD19+ B cells were gated for the following analysis, and CD11c+/high and CD11c- B cells were circled to analyze the frequency and MFI of the positive subpopulation among all immunomarkers, including CD27, CD38, IgD, CD138, T-bet, CXCR5, CXCR3, CD32, and CD21. [file Image_6.tif]

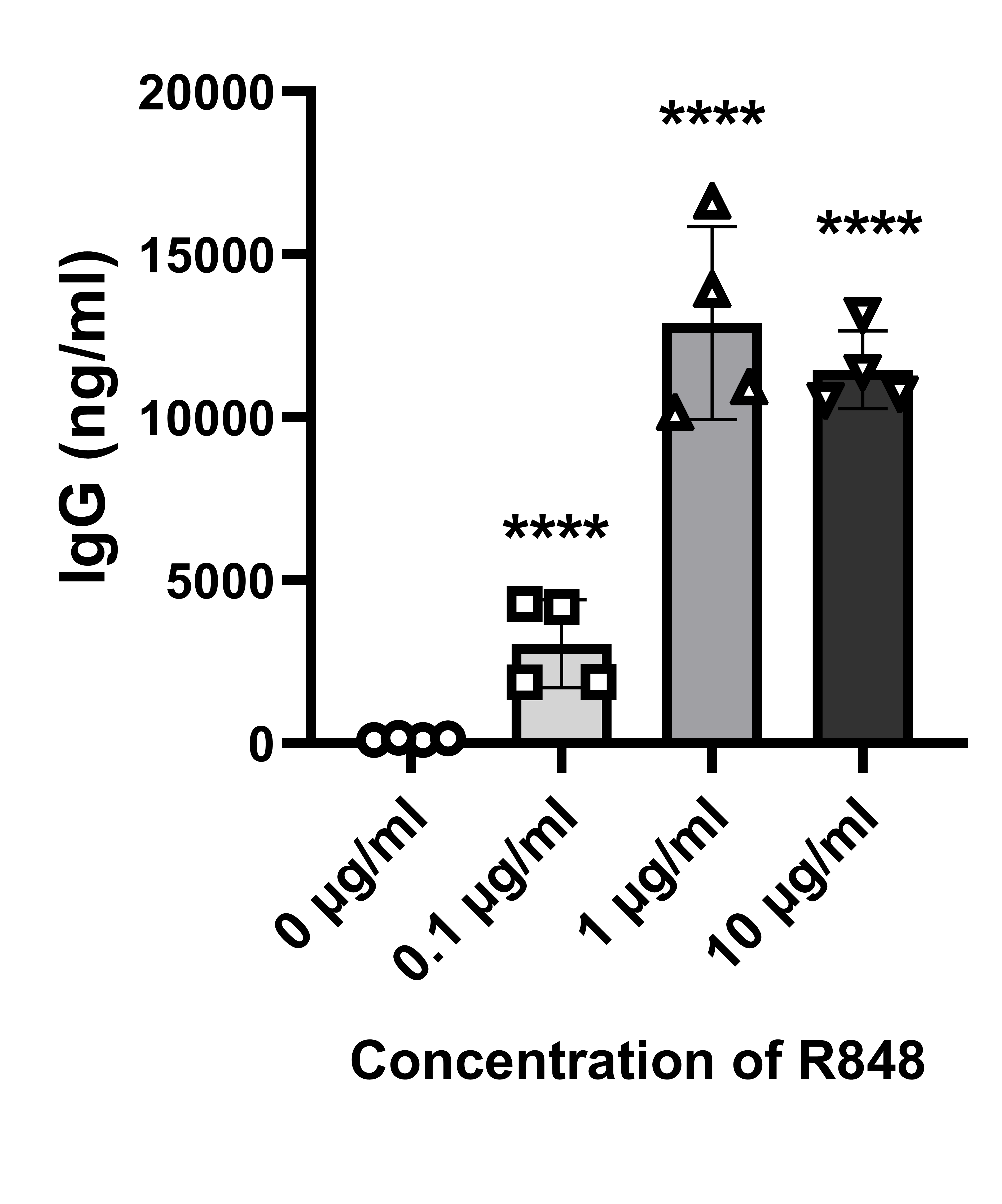

Supplement: Supplementary Figure S7 — IgG production of B cells stimulated with different concentrations of R848. Total B cells from GD patients were stimulated with a concentration gradient of R848 (a TLR7/8 agonist). The culture supernatants were collected on day 9 and measured by ELISA. Data are presented as the mean ± SD and were assessed by ANOVA. The 0.1, 1, and 10 μg/ml groups were compared with the 0 μg/ml group, and the results are marked above the histogram bars. P < 0.05 was considered statistically significant. ns, not significant; ****P < 0.0001. [file Image_7.tif]
