# Supplementary material for: CD11c+ B Cells Participate in the Pathogenesis of Graves’ Disease by Secreting Thyroid Autoantibodies and Cytokines
Source: Front Immunol. 2022 Mar 21;13:836347. doi: 10.3389/fimmu.2022.836347 (PMC8977450; doi:10.3389/fimmu.2022.836347)
Supplement: Supplementary Table S1 — Antibodies used in flow cytometry analysis. [file Table_1.docx]

**Table S1. Antibodies used in flow cytometry analysis.**

| Immunomarker | Fluorochrome | Cat No. | Clone No. | Usage | Company |
| --- | --- | --- | --- | --- | --- |
| CD19 | FITC | 302206 | HIB19 | 5μl/test | BioLegend |
| CD11c | PE | 301606 | 3.9 | 5μl/test | BioLegend |
| CD27 | BV510 | 563092 | L128 | 5μl/test | BD Bioscience |
| CD38 | PerCP | 356621 | HB-7 | 5μl/test | BioLegend |
| IgD | PE-Cy7 | 348209 | IA6-2 | 2μl/test | BioLegend |
| CD138 | APC | 356506 | MI15 | 5μl/test | BioLegend |
| T-bet | BV421 | 644815 | 4B10 | 5μl/test | BioLegend |
| CXCR5 | BV510 | 563105 | RF8B2 | 5μl/test | BD Bioscience |
| CXCR5 | PerCP-Cy5.5 | 356909 | J252D4 | 5μl/test | BioLegend |
| CXCR3 | PE-Cy7 | 560831 | IC6/CXCR3 | 5μl/test | BD Bioscience |
| CD32 | APC | 559769 | FLI8.26 | 5μl/test | BD Bioscience |
| CD21 | BV421 | 566260 | B-ly4 | 5μl/test | BD Bioscience |
