# Supplementary material for: CD11c+ B Cells Participate in the Pathogenesis of Graves’ Disease by Secreting Thyroid Autoantibodies and Cytokines
Source: Front Immunol. 2022 Mar 21;13:836347. doi: 10.3389/fimmu.2022.836347 (PMC8977450; doi:10.3389/fimmu.2022.836347)
Supplement: Supplementary Table S2 — Antibodies used in multiplex immunofluorescence staining. [file Table_2.docx]

**Table S2. Antibodies used in multiplex immunofluorescence staining**

| Immunomarker | Cat No. | Clone No. | Dilution | Company | Fluorescent dye in kit | Excitation wavelength | Emission wavelength |
| --- | --- | --- | --- | --- | --- | --- | --- |
| CD19 | ab134114 | EPR5906 | 1: 400 | Abcam | PPD650 | 640 nm | 660 nm |
| CD11c | ab52632 | EP1347Y | 1: 300 | Abcam | PPD520 | 490 nm | 520 nm |
