# Supplementary material for: CD11c+ B Cells Participate in the Pathogenesis of Graves’ Disease by Secreting Thyroid Autoantibodies and Cytokines
Source: Front Immunol. 2022 Mar 21;13:836347. doi: 10.3389/fimmu.2022.836347 (PMC8977450; doi:10.3389/fimmu.2022.836347)
Supplement: Supplementary Table S3 — The lower limits of detection (LLOD) of all cytokines in the Luminex liquid suspension chip. [file Table_3.docx]

**Table S3. The lower limits of detection (LLOD) of all cytokines in the Luminex liquid suspension chip.**

| Cytokines (Bead region) | Hu.IL-1β. (39) | Hu.IL-1ra. (25) | Hu.IL-2.  (38) | Hu.IL-4.  (52) | Hu.IL-5.  (33) | Hu.IL-6.  (19) | Hu.IL-7.  (74) | Hu.IL-8.  (54) | Hu.IL-9.  (77) |
| --- | --- | --- | --- | --- | --- | --- | --- | --- | --- |
| LLOD (pg/ml) | 0.8 | 1.4 | 1.1 | 0.5 | 0.8 | 1.1 | 0.5 | 0.5 | 0.7 |
| **Cytokines (Bead region)** | **Hu.IL-10. (56)** | **Hu.IL-12 (p70).(75)** | **Hu.IL-13. (51)** | **Hu.IL-15. (73)** | **Hu.IL-17A. (76)** | **Hu.Eotaxin. (43)** | **Hu.Basic.FGF. (44)** | **Hu.G-CSF. (57)** | **Hu.GM-CSF. (34)** |
| LLOD (pg/ml) | 0.9 | 0.9 | 2.1 | 4.2 | 0.2 | 14.6 | 6.8 | 1.1 | 4.5 |
| **Cytokines (Bead region)** | **Hu.IFN-γ. (21)** | **Hu.IP-10. (48)** | **Hu.MCP-1. (53)** | **Hu.MIP-1α. (55)** | **Hu.PDGF-BB. (47)** | **Hu.MIP-1β. (18)** | **Hu.RANTES. (37)** | **Hu.TNF-α. (36)** | **Hu.VEGF. (45)** |
| LLOD (pg/ml) | 19.3 | 6.5 | 6.7 | 2.4 | 1.0 | 1.1 | 1.2 | 3.0 | 0.5 |
